# Research Progress on the Application of Food Colloids in Precise Targeted Delivery of Drugs and Bioactive Compounds

**DOI:** 10.3390/gels11090746

**Published:** 2025-09-17

**Authors:** Yong Guo, Chao Ma, Lianxin Du, Yan Xu, Xin Yang

**Affiliations:** 1 College of Sports Sciences and Health, Harbin Sport University, Harbin 150008, China; guoyong@hrbipe.edu.cn; 2 School of Chemical Engineering and Technology, Harbin Institute of Technology, Harbin 150001, China; mmmmachao1996@163.com; 3 Graduate School, Harbin Sport University, Harbin 150008, China; dulianxin@hrbipe.edu.cn

**Keywords:** food colloids, delivery systems, bioactive food ingredients, bioactive compounds, targeted delivery

## Abstract

With the rapid development of targeted medications and personalized nutritious foods, several bioactive compounds or pharmaceuticals have received a lot of attention for their great functional qualities. However, practical applications confront significant restrictions since these functional compounds frequently exhibit poor solubility and bioavailability during distribution. Food-grade colloidal materials, with their superior biocompatibility and safety profile, have emerged as extremely promising medication and nutrition delivery alternatives. Using food colloidal carrier systems allows for effective targeted drug release while improving the stability and transport efficiency of bioactive compounds. As a result, this study analyzes the design concepts and preparation procedures for food colloidal carriers, as well as outlines research advances in several food colloidal-based tissue delivery systems. Furthermore, this paper discusses the most recent applications of food colloidal systems in delivering unstable bioactive compounds (such as vitamins and minerals) and provides future development possibilities for food colloidal delivery systems.

## 1. Introduction

With growing demand for precision medicine and tailored nutrition, targeted delivery systems have grown in popularity in the biomedical and food science disciplines. Certain naturally sourced food colloids, such as biomacromolecular materials like gelatin, pectin, and carrageenan, have emerged as research hotspots due to their unique rheological properties, biocompatibility, and environmental responsiveness, making them ideal for the development of smart delivery carriers [1,2,3]. Food colloids are micro- and nanoscale dispersed systems generated by the self-assembly of components such as proteins, polysaccharides, and lipids [4,5]. They exhibit functional features such as thickening, emulsification, and controlled release, which improve nutrient bioavailability and allow for precise medication administration [6,7]. Submicron emulsions, colloidal particles, liposomes, and micelles are examples of common food colloids, each with its own structure and function [8,9]. Food colloids have unique physicochemical features and biocompatibility, which can greatly improve medication and nutrient delivery efficiency [10,11]. Leveraging their size features, permeability, and surface qualities allows for steady and tailored delivery of medicines, nutrients, and bioactive substances [12]. For example, the mechanical stability of micelles generated by interfacial self-assembly of colloidal particles, along with surface chemical changes, allows for selective targeting. Concurrently, surface modification of colloidal carriers has emerged as an efficient method for increasing accumulation in target tissues while avoiding biological clearance systems [13]. Within food systems, these colloidal carriers improve the solubility, stability, and intestinal absorption of encapsulated bioactive compounds, hence improving the nutritional and functional aspects of finished products [14,15].

The development of smart food colloidal delivery systems allows for regulated release of natural macromolecules and thermosensitive pharmaceuticals. Non-covalent interactions, for example, can be used to create thermosensitive response systems for natural bioactive compounds, allowing for customizable drug release kinetics and maintaining stability under physiological settings. The customizable degradation and release behavior of emulsion gels can be used to load bioactive proteins and peptides [8,9]. Similarly, food colloidal technology can be used for nutrition delivery. Designing steady-state targeted delivery systems allows for controlled release of functional dietary components, which is essential for nutritional interventions and chronic illness management [16]. Such systems not only increase the bioavailability of bioactive substances, but also modulate their release kinetics in response to physiological circumstances, hence promoting therapeutic and preventive health initiatives [14,16]. Moreover, the utilization of lipid-based colloidal carriers that replicate the “food effect” to improve the oral bioavailability of poorly soluble medicines broadens the applicability of these systems in both food and pharmaceutical sectors [17]. In contrast to conventional synthetic carriers characterized by low bioavailability, inadequate targeting accuracy, and possible toxicity, food-based colloidal delivery systems provide tunable gelling properties and encapsulation stability, as well as modifications responsive to pH, temperature, or enzymes [18,19,20,21,22]. This is essential for improving the release efficacy of active compounds within the complex gastrointestinal environment.

Consequently, the development of efficient and safe targeted drug delivery systems is crucial for enhancing disease treatment outcomes and the efficacy of dietary interventions. This review seeks to encapsulate advancements in food colloidal materials for the precise targeted delivery of pharmaceuticals and nutrients, emphasizing design strategies for food colloidal carriers and their applications in diverse targeted delivery systems, while also delineating prospective stages of development for food colloidal delivery systems.

## 2. Food Colloid Delivery Carriers

Colloidal delivery systems in food can significantly improve the encapsulation, protection, and targeted administration of pharmaceuticals and bioactive substances. Progress in this domain has demonstrated that natural macromolecules and food-grade materials can be employed to regulate essential properties such as solubility, stability, and release kinetics to enhance bioavailability and nutritional efficacy [2,3,23]. These technologies provide considerable benefits compared to conventional delivery methods, facilitating regulated release, minimizing degradation during gastrointestinal transport, and allowing for precise targeting and prolonged release.

Food colloidal delivery systems made of hydrophilic substances such proteins, polysaccharides, and lipids work as encapsulating matrices while also enhancing sensory attributes and prolonging shelf life [4,23]. Casein micelles and milk fat globules, as naturally formed structures, have benefits for biocompatibility and stability owing to their intrinsic capacity to contain and distribute bioactive compounds. Colloidal systems developed only from plant-based components (e.g., lipids, proteins, polysaccharides) offer a sustainable approach to enhancing the solubility and chemical stability of encapsulants [24]. Moreover, emulsion gels can be engineered with particular architectures to regulate the degradation and release characteristics of encapsulated compounds, rendering them suitable for functional food and pharmaceutical applications [1].

Besides these traditional approaches, advanced colloidal structures, including colloids and nano-emulsions, have been thoroughly investigated for their capacity for targeted and sustained delivery. Colloids are generated through the self-assembly of colloidal particles at a fluid interface, exhibiting remarkable mechanical strength and precise permeability control, therefore serving as efficient vesicular carriers for medicinal medicines. Likewise, radical nano-emulsions can promote the dispersion and gastrointestinal absorption of hydrophobic bioactives and pharmaceuticals, thereby considerably improving their bioavailability [25,26]. Moreover, certain biomolecules, including chitosan and sodium alginate, can be utilized in the formulation of colloidal delivery systems [27,28].

Recent advancements in the design of food colloidal delivery systems underscore the interdisciplinary convergence of materials science, nanotechnology, and nutrition. Utilizing the intrinsic characteristics of macromolecules enables the creation of carriers ensuring the stability and tailored release of bioactive compounds while also improving the overall nutritional and sensory attributes of the final product [2,3,23]. Future study may concentrate on enhancing the specificity of delivery systems via improved surface modification techniques and investigating multifunctional platforms that integrate medicinal delivery with targeted dietary tactics.

### 2.1. Definition and Classification of Food Colloids

The phrase “food colloid” refers to multiphase systems where one or more components are scattered as particles, droplets, or bubbles within a continuous media, often ranging from nanometer to micrometer sizes. The definition of “food colloid” systems relies on the interplay between the dispersed and continuous phases, yielding distinctive physical features such as turbidity, viscosity, and the capacity to form organized networks [29,30]. This definition of “food colloid” delineates the raw materials utilized in the preparation of food colloids, encompassing components such as biopolymers, proteins, lipids, and polysaccharides. It also considers the forces that govern the interactions among particles, including electrostatic forces, spatial potential resistance, and van der Waals forces, which affect stability, rheology, and functionality [29,30].

Food colloids can be categorized in multiple ways, primarily according to their physical condition and structural composition. This classification approach categorizes food colloids into systems including emulsions, foams, gels, and dispersions. Emulsions entail the dispersion of one immiscible liquid within another liquid (often oil-in-water or water-in-oil), whereas foams comprise gases dispersed in a liquid or solid matrix. Gels are a category of colloidal systems defined by a three-dimensional network of immobilized continuous phases, exemplified by emulsion gels that incorporate dispersed emulsions inside a gel matrix [1]. The definition and classification of “food colloid” have been further enhanced by ongoing study. Initial research concentrated mostly on the physical characterization and stability of the systems [29]; however, subsequent investigations have broadened the focus to encompass dynamic measurements, interfacial rheology, and the influence of microstructure on macroscopic parameters [30]. Ongoing advancements in research enhance the comprehension and utilization of colloidal interactions, permitting researchers to customize texture, mouthfeel, and shelf-life for the creation of novel food products, while facilitating encapsulation and controlled release of flavors, nutrients, and bioactive compounds [1,29].

In summary, food colloids are characterized by their dispersion structure and interfacial interactions, which are essential to their functional role in food systems. Classification according to composition, physical state, and provenance improves our comprehension of traditional food products and establishes a foundation for innovative technological applications in food design and nutrition. Ongoing advancements in the discipline, propelled by theoretical understanding and practical implementations, affirm that food colloid science is an essential component of study in food engineering.

### 2.2. Overview of Targeted Delivery Systems

Targeted delivery methods are essential for current medication and bioactive molecule administration, improving therapeutic efficacy and reducing systemic side effects. These systems administer pharmacologically active drugs selectively to particular tissues or cells by optimizing the carrier’s characteristics, including size, morphology, surface chemistry, and responsiveness. Initial investigations in this domain concentrated on nanoparticle-based formulations that exploit improved penetration and retention effects in diseased tissues, particularly malignancies [31,32]. Recent advancements encompass carrier design for active targeting via surface functionalization of ligands, antibodies, or peptides to guarantee effective preferential release at the target location [31,33].

Colloidal delivery systems have advanced considerably to include several platforms such as liposomes, polymer nanoparticles, and colloids, each providing distinct benefits regarding stability and controlled release properties. Colloids, created through the self-assembly of colloidal particles at the emulsion interface, exhibit distinctive features like elevated mechanical strength and regulated permeability, enabling tailored release of pharmaceuticals and bioactive substances [6]. The modular design facilitates the creation of multimodal delivery vehicles that can be meticulously adjusted to react to certain biological stimuli or microenvironmental conditions, thereby enabling exact regulation of the amount, location, and timing of drug action [6].

Biocompatible natural materials can be utilized in the formulation of colloidal delivery systems for food products, expanding the scope of targeted delivery applications. For instance, hydrophilic colloids in foods have been utilized to create carriers that safeguard delicate chemicals during gastrointestinal transit while improving their bioaccessibility at the absorption site [23]. Plant-based colloidal systems and tailored emulsion gels have comparable benefits, including excellent biosafety, sustainability, and significant utility in functional food and nutraceutical applications [3,24]. For instance, food colloidal systems consist of proteins derived from soybeans, sunflowers, peanuts, and corn, in conjunction with polysaccharides such as starch, cellulose, and alginates as foundational elements. These devices generally incorporate inherent targeting abilities by adjusting surface charge, hydrophobicity, and receptor affinity to traverse intricate biological barriers [23,24]. Researchers improved the encapsulation effectiveness, photochemical stability, and gastrointestinal release of vitamin D by encapsulating maize zein nanoparticles with carboxymethyl chitosan. Encapsulating curcumin in nanoemulsions enhances its aqueous dispersibility, physical stability, and bioavailability.

Furthermore, the domain has witnessed the advent of design techniques aligned with the principle of Delivery by Design (DbD), which underscores the customization of the physicochemical and biological characteristics of nanoparticle- and microparticle-based systems via a systems approach. These efforts seek to amalgamate concepts from nanotechnology, materials science, and pharmacokinetics to develop carriers that enhance therapeutic indices via passive and active targeting mechanisms [9]. Advanced modifications such as polyethylene glycolization (PEGylation) and ligand coupling can extend circulation duration and improve selective cellular uptake, hence augmenting the efficacy of these targeted delivery systems [9]. Moreover, encapsulation technologies utilizing protein nanocages and other self-assembled biomolecules have created new opportunities for precise targeting, tackling the stability and bioavailability issues related to numerous therapeutic agents [2,34].

The advancement of targeted delivery systems demonstrates the integration of colloidal carrier structural design with biological interfaces to guarantee the controlled and effective distribution of therapeutically active substances. This multidisciplinary approach has expedited progress in drug delivery technology and established a basis for future innovations in precision medicine, where the interaction of carrier characteristics and biological responses is essential for therapeutic success.

### 2.3. The Physicochemical Properties of Food Colloids and Their Application Basis in Delivery Systems

Food colloids are intricate multiphase systems, and their efficacy as delivery carriers is intimately linked to characteristics such as particle size, surface charge, composition, morphology, and reactivity to environmental stimuli [2,35]. Their physicochemical features dictate encapsulation efficiency, stability, controlled release capability, and bioavailability of active ingredients in delivery systems, forming the foundation for their utilization in targeted delivery systems. The dimensions and uniformity of colloidal particles are crucial for the encapsulation and subsequent release kinetics of bioactives at the molecular level. Qiu et al. [35] underscored that meticulous regulation of particle size, composition, and surface functional groups is crucial for ensuring consistent carrier performance. These parameters influence both the level of protection provided to the encapsulated chemicals and their interaction with biological barriers, such as the gastrointestinal mucosa, hence improving absorption [2]. In systems intended for oral administration, optimizing particle size can enhance epithelial absorption while preventing fast evacuation.

Besides size, the surface characteristics of colloidal particles, such as charge density and hydrophobicity, are crucial factors influencing the stability of emulsions and microgels. Dickinson [36] asserted that the system’s general characteristics (e.g., texture and rheology) are dictated by the spatial configuration and physicochemical interactions among proteins, polysaccharides, and other scattered components. Interfacial phenomena are affected by tiny molecules and salts, and can be adjusted by electrostatic interactions and spatial site resistance. Such interactions are crucial in the design of embedded carriers for sensitive bioactives to ensure their protection from adverse environmental conditions prior to reaching the site of action.

The pH sensitivity and environmental responsiveness of specific colloidal carrier systems are fundamental to their use in controlled release applications. Zhang et al. [37] observed that alginate hydrogel beads have pH-dependent stability, protein retention characteristics, and release kinetics, which are essential for preserving the integrity of encapsulated proteins during transit in fluctuating pH environments, such as the stomach and intestine. This responsiveness enables the carrier to deliver the active ingredient in a regulated fashion, guaranteeing optimal compound bioavailability at the target location. This tailored release profile addresses the constraints associated with the degradation of sensitive biomolecules.

The adaptability of colloidal systems is shown in the creation of tailored colloids that concurrently improve sensory attributes and functional advantages in food matrices. Douaire and Norton [38] observed that altering the microstructure of a colloid (e.g., by creating a structured network or an emulsion gel) might influence attributes such as mouthfeel, flavor release, and satiety. These qualities are associated with consumer acceptance and substantially enhance the efficient administration of nutraceuticals and pharmaceuticals. The relationship between the structural modification of colloids and their functional characteristics in delivery applications establishes a robust basis for their utilization in diverse culinary and medicinal systems [38].

The physicochemical features of food colloids, such as particle size, surface functionalization, interfacial behavior, and pH responsiveness, are critical factors in the development of efficient delivery systems. These qualities allow carriers to encapsulate, safeguard, and release bioactive chemicals in a regulated way, hence improving their therapeutic efficacy and functional performance in food and pharmaceutical applications. Progress in colloid science is expanding the use of these systems, facilitating creative approaches for precision nutrition and tailored medicines.

#### 2.3.1. Rheological Properties of Food Colloids

The rheological characteristics of food colloids are essential for comprehending their processing behavior and the sensory attributes of the final product. The properties, such as viscosity, shear thinning, yield stress, and viscoelastic modulus, result from the intricate interactions among dispersed particles, the continuous phase, and the particles inside the system [39]. The rheological behavior influences the stability and processing characteristics of food colloids, including emulsions, gels, and foams, and is directly associated with the texture and mouthfeel experienced by consumers.

**Figure 1:**
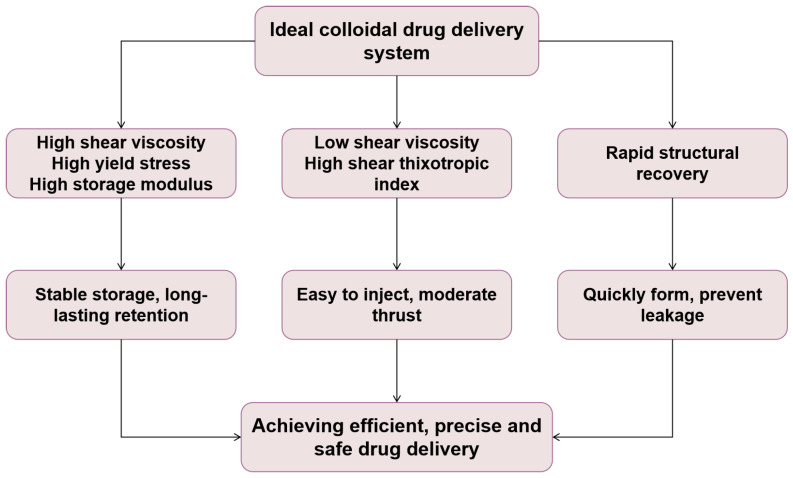
Schematic Diagram of the Influence of Rheological Characteristics on Materials.

A fundamental aspect of rheological investigations is the characterization of the viscoelastic characteristics of food colloids in reaction to applied stress. In the process of ice cream manufacturing with whipped toppings, the stalling of viscoelastic fat aggregation results in the creation of trans-volumetric networks. These networks improve the product’s structure and stability, while influencing its flow characteristics and sensory attributes [40]. The microstructure of the colloidal spatial configuration has been associated with macroscopic rheological characteristics, including energy storage modulus and energy dissipation modulus [41]. Utilizing these not only enhances the texture of the final product but also generates innovative concepts for the design of new soft materials with customized rheological characteristics. The colloidal rheological characteristics affect the material as shown in Figure 1.

Furthermore, environmental parameters including temperature, ion type, and ionic strength significantly influence the dynamic and steady-state rheological properties of colloidal dispersions. Research on the rheological properties of Honeybush seed gum dispersions indicates that variations in temperature and electrolyte concentration can markedly influence the steady-state shear behavior and dynamic viscoelasticity of these systems [42]. Exact regulation of rheological properties during manufacturing processes (e.g., pumping, spraying, or 3D printing) guarantees optimal processing efficiency and product quality.

Under oscillatory shear, the rheology of food colloids demonstrates intricate phenomena, including two-step yielding and strain-induced directional strengthening. The destruction and subsequent re-formation of the microstructural network under oscillatory shear results in a nonlinear rheological response [43]. Minor alterations in the microstructure (e.g., aggregation or network connection) might result in substantial modifications in the macroscopic rheology. These are crucial for forecasting product behavior in dynamic processing operations and for creating resilient models that align with microstructural and macroscopic rheological characteristics.

In conclusion, the multiscale coupling of particle interactions, network development, and external processing conditions constitutes the rheological foundation of food colloids. A thorough comprehension of these connections can improve the design of food colloid systems and assist in customizing textural and sensory attributes to fulfill customer expectations. The intricate dynamics of these linkages may be leveraged in the future to innovate new goods and enhance conventional food processing.

#### 2.3.2. Stability and Encapsulation Capacity of Food Colloids

In recent years, food colloids have garnered significant attention as efficient carriers for the encapsulation and stabilization of bioactive chemicals. Two major performance measures for these systems are encapsulation capacity (i.e., the efficacy of retaining bioactive chemicals) and stability under diverse storage and processing settings.

Numerous studies indicate that the meticulous formulation of food colloids can markedly enhance their encapsulation effectiveness and durability. Nanoliposomes engineered to encapsulate grape seed tannins exhibited a high encapsulation efficiency (~79% for epicatechin), demonstrated exceptional physicochemical stability for up to 90 days under refrigeration, maintained a controlled particle size distribution, and possessed an appropriate surface charge (zeta potential −41.6 mV) [44,45]. Alginate-based capsules utilized for encapsulating Lactobacillus plantarum and beet extracts attained encapsulation efficiencies ranging from 86.4% to 88% and did not negatively impact the rheological properties of the model food system [45]. These findings indicate that optimal loading capacity and stability can be attained with the appropriate selection of biopolymer and encapsulation technique.

Encapsulation can be enhanced by creating composite nanoparticle systems that offer multifunctional advantages. Lactoferrin-based ternary composite nanoparticles have been engineered for curcumin delivery, attaining high encapsulation efficiencies (~86%) and loading capacity, with enhanced thermal, photostability, and storage stability of curcumin [46]. Core–shell nanoparticles, utilized for astaxanthin encapsulation, offer an efficient approach to enhance water solubility and safeguard sensitive components from degradation in adverse situations [47]. Consequently, the stability of bioactives in vivo can be enhanced by adjusting the core–shell mix, particle size, and surface characteristics.

Besides nanoparticle systems, microemulsion gel systems are extensively utilized for their benefits, such as high solubility and thermodynamic stability, along with the adjustable rheological characteristics of the gel matrix [48]. These solutions can attain elevated encapsulation efficiency and prolonged release profiles while preserving structural stability. For instance, encapsulating lipophilic chemicals like turmeric oleoresin within gelatin-starch matrices, utilizing an emulsification technique to create colloids and incorporating functional additives such as silica, can markedly enhance encapsulation effectiveness and product yield [49].

Nano-emulsions exemplify food colloids that facilitate the effective distribution of bioactive substances. Their diminutive droplet size enhances bioavailability and absorption while safeguarding against extrinsic variables such as light, heat, and oxidation [50]. The stability of encapsulation systems is intricately linked to their microstructure. Emulsion gels formulated for β-carotene encapsulation demonstrate that the interaction between polysaccharides and proteins at the oil–water interface governs droplet morphology and the integrity of the gel network [51]. The physical qualities directly influence the in vitro digestive behavior and bioaccessibility of encapsulated nutrients.

The utilization of biopolymers for the fabrication of nanoparticles to encapsulate polyphenolic chemicals and antioxidants is a novel strategy to enhance stability and bioavailability. The application of biopolymers as carriers enhances the inadequate water solubility and pH sensitivity of encapsulated molecules, hence maintaining the bioactivity of unstable chemicals during storage and digesting [52]. The advancement of tailored colloidal carriers is enhanced by systematic design methodologies, such as the “delivery by design” strategy, which encompasses the optimization of encapsulation and stability parameters, as well as the assurance of targeted delivery and controlled release within intricate food matrices [53].

In conclusion, the encapsulation capacity and stability of food colloids are influenced by various parameters, including particle size, surface charge, microstructural organization, and the selection of biopolymer materials and production methods. Techniques including nanoliposomes, microcapsules, composite nanoparticles, microemulsion gels, and nanoemulsions enhance encapsulation efficiency and stability, thereby augmenting the bioactivity and functionality of bioactive compounds in food and nutraceutical applications [3,44,45,46,48,50].

#### 2.3.3. Interaction Mechanisms Between Food Colloids and Drugs/Nutrients

Comprehending the interactions between food colloids and medications or nutrients is essential for maintaining the stability of delivery systems. In these systems, several noncovalent interactions (e.g., hydrophobic interactions, hydrogen bonding, van der Waals forces, and electrostatic attraction) are crucial for the encapsulation and release kinetics of active ingredients.

A significant method is donor-acceptor ligand interaction, wherein drug loading capacity can be augmented through the use of specific ligand interactions. Polymeric micelles with electron acceptor groups can be conjugated to pharmaceuticals like Adriamycin, resulting in exceptionally high drug loading efficiencies [54]. This method emphasizes the significance of engineering polymer architectures capable of establishing particular interactions with drug molecules, facilitating the effective sequestration of hydrophobic medicines inside the hydrophilic matrix.

Protein-derived colloids, such as zeinolysin nanoparticles, exhibit distinctive pH responsiveness and electrostatic interaction properties in food delivery systems. Corn alcohol soluble protein, a hydrophobic protein sourced from maize, can be modified into nanoparticles that encapsulate chemotherapeutic agents like ellipticine. Pourhossein et al. [55] revealed that electrostatic interactions between the medication and the carrier were diminished in acidic circumstances (pH 5.5), resulting in an increased release rate.

The connection between the medicine or nutrition and the protein-based carrier was augmented by the use of natural proteins such bovine serum albumin (BSA), which enhances colloidal stability and offers various binding sites for the active compound. Neves et al. [56] found that the functionalization of Fe_3_O_4_ nanoparticles with BSA enhanced the affinity and binding strength for salicylic acid, hence boosting encapsulation efficiency and controlled release characteristics. Protein-drug interactions are typically regulated by a combination of hydrogen bonding and hydrophobic interactions, which are crucial for preserving the structural integrity of the colloidal matrix.

A distinct contact mechanism was clarified by colloidosomes, which are hollow microcapsule structures made of colloidal particles. Dinsmore et al. [57] indicated that colloidosomes regulate permeability and elasticity, facilitating the encapsulation of many active compounds, including medicines, proteins, and vitamins. The self-assembly of colloidal particles at the interface creates a resilient shell layer, with their interaction with encapsulated components primarily influenced by interfacial processes, including hydrophobic interactions and surface adsorption.

Emulsion gels, generally formulated from food-grade proteins and polysaccharides, depend on several interaction mechanisms for encapsulation and controlled release. Abdullah et al. [1] presented a thorough examination of protein-polysaccharide interactions for the formation of gel network structures, stabilization of physiologically active substances, protection against degradation, and regulation of their release during digestion. Rajić et al. [58] illustrate that maize alcohol-soluble protein-resin composite nanoparticles exploit interactions between alcohol-soluble biopolymers and hydrophobic bioactive compounds, with compatibility between the biopolymer matrix and the active ingredient being augmented by hydrophobic interactions. Perry and McClements [2,59] highlighted that the effective design of these colloidal systems depends on the customization of molecular interactions. These interactions are methodically utilized to enhance loading capacity, protective characteristics, and tailored release profiles by adjusting parameters such as particle size, surface charge, and hydrophobicity. Biopolymer-based nanostructures generally employ electrostatic interactions to establish stable complexes with charged pharmaceuticals, but hydrophobic interactions are essential for encapsulating lipophilic substances.

Alternative techniques, such as layer-by-layer (LbL) construction, further exemplify the significance of electrostatic and hydrogen bonding interactions. Połomska et al. [60] examined the utilization of LbL coating technology, which offers a flexible approach to adjust the permeability of colloidal carriers by successively applying oppositely charged polymers around the drug core to facilitate controlled release and improve colloidal stability.

Moreover, stimulus-responsive methods have been utilized in the design of colloidal carriers to modulate the release of medications or nutrients in response to alterations in the external environment. Liu et al. [61] engineered Pickering emulsions capable of activating “nanogates” upon UV irradiation, showcasing programmable release functionalities. This method integrates interfacial stability with light-induced conformational alterations in the colloidal shell layer, so offering a novel means to externally regulate release kinetics.

In conclusion, the interaction processes between food colloids and medications or nutrients are intricate and encompass several complex interactions, including non-covalent interactions. Colloids are formulated and synthesized through interactions including donor-acceptor coordination, pH responsiveness, electrostatic interactions, protein-drug binding, and interfacial self-assembly, which influence encapsulation efficiency, colloidal stability, and the release kinetics of active ingredients. A comprehensive understanding of the fundamental interaction mechanisms has not only propelled the construction of precision-targeted delivery systems but also fostered innovation in the wider domain of functional food and medicine delivery.

#### 2.3.4. Interfacial Behavior Has a Decisive Impact on Delivery Efficiency

Interfacial behavior is crucial for the distribution efficiency of colloidal carriers by influencing the encapsulation, stability, and controlled release of medicines and nutrients. The characteristics of the interfacial layer (e.g., interfacial tension, viscoelasticity, adsorption kinetics, and dynamic rearrangement of colloidal particles) directly affect the release of bioactive chemicals in the physiological environment.

Microscale interfacial tension measurements are crucial for elucidating the regulatory processes underlying droplet stability and encapsulation efficiency. Research on micrometer-scale droplets indicates that reduced interfacial tension facilitates the development of stable emulsions, crucial for preserving the integrity of encapsulated actives throughout storage and digesting [62]. In Pickering emulsions, where solid particles are adsorbed at the oil–water interface, the orientation and stiffness of the interfacial layer are especially crucial. Wang et al. [63] discovered that zeinolysin/chitosan composite particles can create a resilient membrane layer at the interface, which not only bolsters oxidative stability via a physical barrier but also augments the efficiency of interfacial antioxidant delivery, thereby enhancing overall bioefficacy.

Research in microfluidics has underscored the significance of interfacial dynamics in double emulsions. Chen et al. [64] observed that altering the viscoelastic properties of the interfacial coating through the addition of chemicals like alginate can directly influence emulsion stability. Augmented interfacial elasticity enhances deformation resistance and diminishes agglomeration, which is crucial for ensuring the regulated release of encapsulated chemicals. Jin et al. [65] discovered that protein-polysaccharide nanocomplexes can irreversibly modulate droplet size and wettability at the interface, hence facilitating precise regulation of nutrient release profiles.

The configuration of proteins at the interface significantly influences the overall efficacy of the delivery system. Proteins, owing to their amphiphilic characteristics, spontaneously adsorb at the oil–water interface, creating an adsorbent layer that offers electrostatic and spatial positional stabilization resistance. Nik et al. [66] observed that the design of protein-stabilized emulsions directly influences the interfacial composition during digestion, thereby affecting the bioaccessibility and absorption efficiency of nutrients. A study by Macierzanka et al. [67] demonstrated that the structural structure of the adsorbed protein layer influences interfacial protein hydrolysis and its resistance, hence directly impacting the release kinetics of nutraceuticals and medicines in the gastrointestinal tract.

Investigations of multiscale interfacial structures have enhanced the overall comprehension of these phenomena. Marze [68] highlighted that the phase of the body and interfacial configuration in dispersed food systems collectively influence the bioaccessibility of micronutrients, indicating that precisely adjusted interfacial characteristics can markedly improve delivery efficiency. Xiao et al. [69] emphasized that the equilibrium of colloidal particles at the interface of food-grade Pickering emulsions, along with wettability and stacking patterns, is essential for attaining superior stability against aggregation and delamination, thereby ensuring the intact delivery of active compounds.

The capacity of food colloids to adsorb at the oil–water interface directly influences the stability of the emulsion system. The synergistic interaction between whey protein and inulin diminishes interfacial tension and creates a more robust interfacial layer, hence enhancing the retention of vitamin D3 during digestion. In vitro simulated digestion trials shown that xanthan gum impeded stomach emptying by enhancing system viscosity, leading to a 40% increase in the fraction of insulin designated for intestinal release. Colloid-biofilm interactions significantly influence cellular absorption efficiency. Carboxymethylcellulose-modified nanoparticles facilitate the absorption of quercetin by intestinal epithelial cells via lattice protein-mediated endocytosis.

In conclusion, the interfacial characteristics of colloidal carriers, encompassing interfacial tension, adsorption kinetics, and viscoelastic film formation, are critical determinants of delivery effectiveness. Refining these interfacial parameters via meticulous design and material selection can improve stability, encapsulation capacity, and controlled release characteristics of medicines and bioactive nutrients, hence augmenting their bioavailability and therapeutic effectiveness.

### 2.4. Common Food-Grade Colloidal Materials and Their Characteristics

Food-grade colloidal materials serve as essential components in the development of sophisticated delivery systems for bioactive substances, with their inherent features critically influencing encapsulation efficiency, stability, and release patterns. These colloids, originating from natural biomolecules like proteins, polysaccharides, and lipids, are frequently transformed into nanoparticles, emulsions, gels, and Pickering systems. Their biocompatibility, repeatability, and adjustable interfacial characteristics render them appropriate for food applications [29,62].

Proteins are the most widely used food-grade colloidal materials. Common proteins include soy protein, pea protein, ovalbumin, and whey protein. Many proteins are amphiphilic molecules that can be used to encapsulate hydrophilic and hydrophobic substances. For instance, naturally occurring systems like casein micelles and milk fat globules in milk can encapsulate and safeguard hydrophobic compounds, serving as a model for engineered systems; likewise, legume and flaxseed proteins have been examined for their superior emulsification, foaming, and film-forming capabilities, which are closely linked to their particle sizes and substantial water-absorption capacity. Furthermore, nanoparticles derived from complex proteins (e.g., lactoferrin ternary system) exhibit improved dispersibility and superior preservation of hydrophobic nutrients like curcumin, hence mitigating issues related to poor water solubility and low bioavailability [2,46].

Emulsion gels are structured colloids assembled from proteins and polysaccharides to provide three-dimensional networks capable of trapping large amounts of water, oil, or air, combining the functional advantages of controlling texture and mouthfeel, as well as having the ability to encapsulate bioactive compounds [1]. In addition, microemulsion systems characterized by thermodynamic stability and fine droplet size are used to solubilize and protect sensitive bioactive, which are often stabilized with natural surfactants and biopolymers chosen to meet food-grade standards [65].

Stabilized for consumption Pickering emulsions derived from natural raw materials have distinctive interfacial characteristics owing to their irreversible adsorption at the oil–water interface. Recent research indicates that food-grade particles, such as those derived from plant saponins (including saponin and glycyrrhizic acid) or protein aggregates, effectively stabilize Pickering emulsions, augmenting resistance to agglomeration and strengthening encapsulation stability [66,67,68,69]. The utilization of colloidal systems relies on comprehending the interfacial mechanisms governing adsorption, particle aggregation, and viscoelastic properties, which collectively influence the efficacy of these delivery systems.

**Figure 2:**
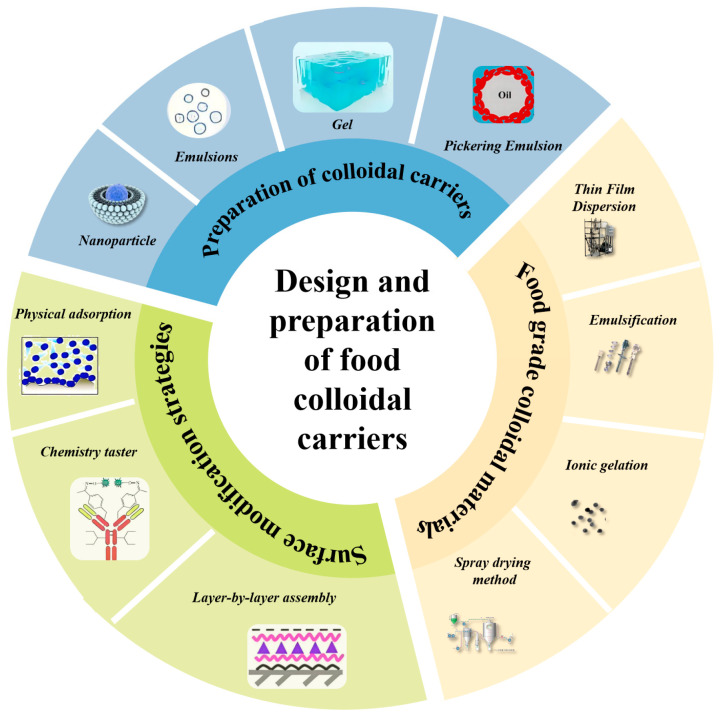
Preparation of colloidal carriers.

Common food-grade colloidal materials, including proteins (e.g., casein, legume proteins, and flaxseed proteins), polysaccharide-protein complexes, and plant-derived saponins, demonstrate unique physicochemical properties such as elevated interfacial activity, emulsification potential, and the capacity to create structured networks. These features are fundamental to the customization of food colloids with specific functionalities, employed to regulate micronutrient release and boost bioavailability, as well as to improve the texture and shelf life of functional foods. Food-grade colloidal systems are sustainable and biocompatible, now employed in various applications including nutraceuticals and medicine administration, as seen in Figure 2.

### 2.5. Preparation Methods of Colloidal Carriers

Diverse techniques for the synthesis of colloidal carriers for food and nutraceutical applications have been established, employing both bottom-up and top-down approaches. Preparation procedures are chosen according to the enclosed substance, intended release profile, and scalability requirements. Researchers have devised methods from traditional emulsification and coalescence to sophisticated techniques including nanoprecipitation, spray drying, freeze drying, electrostatic spinning, and layer-by-layer (LbL) assembly to guarantee that the resultant carriers uphold food-grade safety and functionality [70,71,72].

Emulsification is a prevalent technique for encapsulating lipophilic functional food components (e.g., taste compounds, vitamins, and essential oils) by the preparation of micro/nanoemulsions. For instance, the nanoencapsulation of citrus essential oils was accomplished by formulating oil-in-water systems that utilize natural surfactants and biopolymers to provide thermodynamic stability and facilitate controlled release features [73,74]. Emulsification procedures often employ high-energy methods, such high-shear mixing, sonication, or homogenization, to diminish droplet size and create stable emulsions. Furthermore, double emulsions have been engineered to encapsulate multiple bioactives simultaneously; for instance, trans-resveratrol and vitamin D3 can be incorporated into concentrated double emulsions concurrently, enhancing their drug-carrying capacity and stability across various environments [75]. Nevertheless, the emulsification technique has obstacles including inconsistent dispersion phase size, a propensity for emulsions to separate, and a limited shelf life.

Alternative techniques, like complex coalescence and nanoprecipitation, facilitate the generation of colloidal carriers from biopolymers, often proteins and polysaccharides. In these processes, electrostatic interactions or solvent exchange among biopolymers can facilitate self-assembly and phase separation, resulting in the creation of particles with a regulated core–shell structure. Sodium caseinate-stabilized zeinolysin particles can be synthesized via an anti-solvent precipitation method, wherein the intrinsic amphiphilicity of casein and zeinolysin facilitates the creation of natural food-grade colloidal systems for encapsulating hydrophobic nutraceuticals [76]. The utilization of sodium alginate and guar gum facilitates the encapsulation of chemicals like streptozotocin lactate, thereby safeguarding and regulating the release of antimicrobial agents from food matrices [77].

Spray drying and freeze drying are techniques specifically designed for the creation of microencapsulation. These techniques facilitate the fast elimination of solvents to produce powders, thereby encapsulating the bioactive substances within the biopolymer matrix. These drying methods are extensively employed in the encapsulation of polyphenolic compounds and vitamins, with freeze-drying being especially effective in maintaining the integrity of heat-sensitive constituents; however, challenges such as particle aggregation and bioactive degradation post-drying persist [78,79]. The selection between spray drying and freeze drying typically hinges on the balance of production costs, particle shape, encapsulation efficiency, and overall stability.

**Figure 3:**
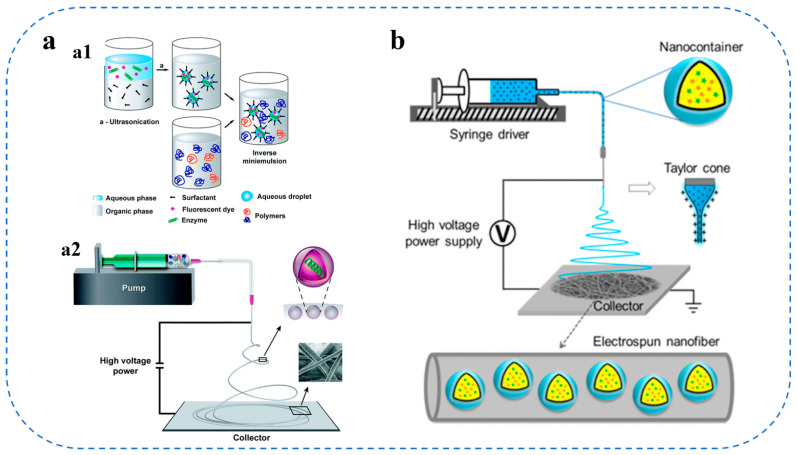
Preparation Methods of Colloidal Carriers. (**a**) Preparation of nanofibers using microemulsion-electrospinning technology [80]; (**b**) Electrostatic spinning method for the preparation of colloidal containers with colloidal fibers [81].

Advanced methods, including electrostatic spinning and fine-emulsion electrostatic spinning, have been investigated for the fabrication of nanofiber carriers for the production of loaded bioactives. Poly(ester amide) nanofibers for amino acids were synthesized using a refined emulsion-electrostatic spinning technique, wherein encapsulated enzymes were preserved in submicron compartments, as illustrated in Figure 3a, resulting in scaffolds with customized enzyme degradation characteristics for controlled release applications [80]. Furthermore, by integrating colloid-based encapsulation with electrostatic spinning, the nanocontainers can be incorporated into or onto the fiber matrix [81]. These methodologies can be utilized in research on active food packaging that necessitates the regulated release of preservatives or antioxidants. However, electrospinning technology has relatively low production efficiency and struggles to meet the demands of large-scale production.

Liposome encapsulation techniques represent a significant category of methods for the creation of colloidal carriers. Liposomes, composed of phospholipid bilayers, are generally formed using thin-film hydration, subsequently followed by sonication or extrusion to yield vesicles with a regulated size distribution and surface charge. These vesicles are engineered to extend circulation duration and improve stability through alterations in lipid composition and surface characteristics, as depicted in Figure 3b [82,83]. Conventional liposome production techniques are frequently utilized in pharmaceutical research; however, their use has now extended to encapsulating bioactive proteins and peptides into functional food systems for nutraceutical applications [2].

Solid lipid nanoparticles (SLNs) are an alternative formulation approach that employs the solid state of the lipid matrix at ambient temperature for regulated release. For instance, SLNs containing ergocalciferol can encapsulate fat-soluble vitamins, so decelerating digestion and facilitating prolonged release throughout the gastrointestinal system [84]. The lipid core’s hydrophobicity and the presence of surfactants can sequester bioactives in adverse conditions, enabling solid matrices to reduce diffusion and avert premature release.

Ultimately, composite and multilayer compositions enhance the adaptability of colloidal carriers. The layer-by-layer (LbL) assembly technique facilitates the successive application of oppositely charged biopolymers around a core, creating a protective shell that regulates permeability and stability [72]. This method enables the integration of various functionalities within a single particle, suitable for the targeted administration of vitamins, antioxidants, and other nutrients [85].

In summary, the formulation of colloidal carriers for food applications is complex and depends on several encapsulation methods. Emulsification (including single and double emulsions), coagulation, nanoprecipitation, spray and freeze-drying, liposome formation, solid lipid nanoparticles (SLNs), and sophisticated methods such as electrostatic spinning and layer-by-layer (LbL) assembly offer distinct benefits for attaining elevated encapsulation efficiency, stability, and controlled release.

### 2.6. Surface Modification Strategies for Colloidal Carriers

Surface modification of colloidal carriers is a crucial approach to improve their stability, targeting efficacy, circulation duration, and overall therapeutic effectiveness. Surface changes can be accomplished using covalent and non-covalent processes, including physical adsorption, chemical coupling, layer-by-layer assembly, and customization of carrier interface attributes. These alterations can alter surface charge and hydrophobic/hydrophilic equilibrium, enhancing the attachment of certain ligands or stealth polymers, thus boosting interactions with biological systems [86].

A variety of chemical modification techniques have been established to impart unique functionalities to colloidal carriers. Colloids have been synthesized through incremental surface modification of natural minerals, such as elohimite nanoclay, utilizing Janus colloidal surfactants characterized by different zones exhibiting opposing wettability [87]. This method integrates the benefits of molecular surfactants with the stability of Pickering emulsions, facilitating tailored drug administration. The association of proteins with polysaccharides to create biocouples constitutes another feasible approach. Protein and polysaccharide couplings have emerged as attractive scaffolds for drug delivery owing to their great biocompatibility and degradability, while selectively attaching medicinal molecules [88]. When altering food colloidal carriers using chemical modification procedures, it is essential to evaluate the safety of the reagents and the potential impact of the inserted functional groups on the functional activity of the natural compounds.

**Figure 4:**
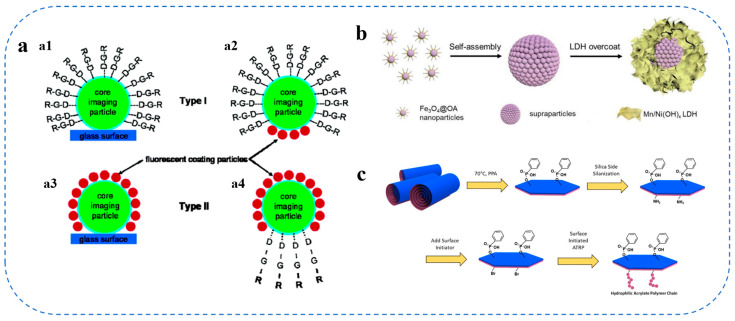
Surface Finishing Strategies (**a**) Localized functionalization of individual colloidal carriers [90]; (**b**) Construction of bilayer colloidal carriers [94]; (**c**) Development of Janus Nanoplate Surfactants for the Formation of Stabilized Pickering Emulsions [87].

Alongside chemical coupling, physical methods like layer-by-layer (LbL) assembly have become increasingly common. The LbL approach facilitates the successive adsorption of oppositely charged polymers or biomolecules, resulting in the formation of multilayer shells with regulated permeability and release characteristics [89]. Moreover, localized surface functionalization techniques have been employed to attain spatially regulated ligand presentation, enhancing cell targeting and intracellular imaging capabilities, as illustrated in Figure 4a [90,91]. This method enables the customization of distinct areas on colloidal carriers for targeted biological interactions, hence regulating cellular uptake and endocytosis mechanisms [91]. In the formulation of food colloidal systems, changes in purity, molecular weight, and charge density across batches are substantial. The repeatability of assembly is inadequate when utilizing raw ingredients like chitosan and sodium alginate. As a result, the spectrum of accessible electrolytes for formulation via LBL technology is somewhat limited.

The specificity of targeting can be augmented by attaching the ligand component to the carrier surface. Carbohydrate coating and folate functionalization are employed to target lectin receptors or folate receptors on cancer cells [92]. These alterations enhance the affinity for the target tissue and augment the selectivity of drug accumulation at the intended site of action, thus diminishing systemic toxicity and off-target effects [86]. Furthermore, the use of biocompatible polymers like polyethylene glycol (PEG) is frequently employed as a stealth mechanism to circumvent immunosurveillance, thus extending in vivo circulation duration.

Inorganic colloidal carriers also advantage from advanced surface engineering techniques. Methods that incorporate surface functionalization and biocoupling, including the modification of nanoparticles like gold or semiconducting materials with diverse chemical and biological reagents, enhance their utility in imaging, sensing, and targeted therapeutics [93]. Figure 4b illustrates that the incorporation of layered double hydroxides, which emulate oxidative enzymes, onto colloidal platforms has augmented the multifunctional characteristics of these carriers, including catalytic efficacy for antibacterial and wound healing applications [94].

Diverse surface modification strategies, including the preparation of Janus particles (illustrated in Figure 4c [87]), rational engineering of surface charge and hydrophobicity [86], layer-by-layer (LbL) assembly and localized functionalization [89], and the coupling of targeted ligands such as carbohydrates and folic acid [92], indicate that customizing the interfacial properties of colloidal vectors is essential for enhancing their biological interactions and delivery efficiency. These sophisticated alterations can mitigate conventional delivery problems and enhance the evolution of precision-targeted systems for pharmaceutical applications [86,88,89].

## 3. Research Progress on Food-Based Colloidal Systems for Precise Targeted Delivery in Different Tissues

Food colloids have developed into precision-targeted delivery methods to improve the bioavailability, stability, and controlled release characteristics of medicines and nutraceutical bioactive substances. These methods employ natural macromolecules (proteins, polysaccharides, and lipids) to create colloidal carriers, designed for selective targeting and enhanced absorption, addressing the issues of low water solubility and rapid metabolism of numerous bioactive compounds [3,23].

Food-grade colloidal carriers, such as nanoemulsions, liposomes, and emulsion gels, possess intrinsic advantages owing to their biocompatibility and Generally Recognized As Safe (GRAS) status. For instance, nanoemulsion formulations have effectively encapsulated hydrophobic nutraceuticals to enhance aqueous dispersion and gastrointestinal absorption while safeguarding the active ingredients from degradation [38,95]. Emulsion gels offer controlled release conditions and can be tailored to adjust texture and sensory attributes, enhancing customer options and increasing product adoption [1]. The encapsulation procedure generally employs hydrophilic colloids and polymers to facilitate the targeted distribution of bioactives to particular locations within the gastrointestinal system by regulating release kinetics and interactions with the food matrix [23,96].

Moreover, oral cell-targeted delivery methods formulated from consumable components have propelled the advancement of these delivery platforms. They employ the binding properties of particular ligands and carriers to cellular receptors to augment the absorption of encapsulated substances at the intended site of action and minimize off-target effects. Food-grade polymers can be chemically altered to provide controlled-release formulations that ensure prolonged therapeutic effects and reduce negative effects by facilitating the progressive release of bioactive compounds [97]. Polymer systems designed for colon-targeted administration safeguard the encapsulated bioactives from traversing the upper gastrointestinal tract, facilitating their release at designated areas within the colon to address localized gastrointestinal illnesses.

Progress in nanofabrication and surface modification technologies (e.g., layer-by-layer assembly, nanomicelle production) has improved the accuracy of targeted systems. The manipulation of particle size, surface charge, and hydrophobicity in colloidal carriers improves targeting efficacy, maintains transport stability, and facilitates binding and internalization by target cells [98,99]. Temperature-responsive colloidal particles, formed from non-covalently interacting small molecules, provide the potential for stimulus-induced release under physiological settings [5].

The interdisciplinary integration of materials science, food chemistry, and nanotechnology has facilitated the development of intricate food colloidal delivery systems that allow for exact targeting. These technologies enhance the shelf-life and sensory attributes of functional meals while also augmenting the therapeutic efficiency of encapsulated pharmaceuticals and nutraceuticals, presenting considerable potential for preventive nutrition and clinical applications (Table 1).

### 3.1. Colloidal Delivery Systems for Anticancer Drugs

Colloidal delivery methods exploit the tumor-specific milieu to improve the targeted delivery of anticancer pharmaceuticals. These systems have been engineered to enhance the solubility, stability, and bioavailability of chemotherapeutic drugs while minimizing systemic toxicity by precision targeting. Diverse ways have been formulated to employ food-grade colloids or synthetic counterparts, encompassing attributes such as stimulus responsiveness, ligand-mediated targeting, and intelligent release patterns.

Aptamer-based systems represent a potential methodology for precise targeting. These methods employ oligonucleotide aptamers that identify the overexpression of particular receptors on tumor cells to facilitate spatial and trigger-responsive drug release control [100]. Besides aptamers, micellar systems composed of amphiphilic polymers exhibit significant potential. Micelles can be engineered for acidic tumor microenvironments or external stimuli (e.g., light, temperature) to boost drug penetration, extend circulation duration, and promote selective accumulation via enhanced permeation and retention (EPR) effects [101,102]. These micelles can be additionally functionalized with targeted ligands to enhance specific effects on tumor tissues.

**Figure 5:**
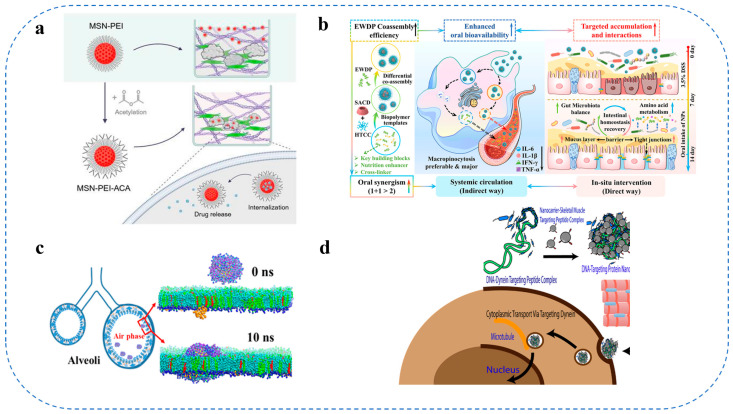
Food Colloids in Precision Targeted Delivery Systems (**a**) Mesoporous silica nanoparticles for targeted cancer therapy [121]; (**b**) Programmable food-derived peptide assembly strategies for targeted colitis therapy [111]; (**c**) Lipid-encapsulated gold nanoparticles for lung-targeted delivery [130]; (**d**) Development of a 5th generation polyamidoamine dendrimer functionalized with the skeletal muscle targeting peptide ASSLNIA (G5-SMTP) [133].

Alternative colloidal systems, including functionalized multi-walled carbon nanotubes (f-MWNTs), employ non-covalent π-π stacking interactions to encapsulate hydrophobic pharmaceuticals (e.g., paclitaxel), while the incorporation of folic acid or poly(ethylene glycol) modifications improves their solubility, biocompatibility, and tumor-targeting efficacy [103]. Degradable polymer nanoparticles, such as polylactic acid-hydroxyacetic acid copolymers (PLGA), passively target tumor tissues via the EPR effect and can regulate drug release through pH-responsive mechanisms [104]. This category of formulations encompasses surface-modified mesoporous silica nanoparticles (MSNs) utilized as high drug-loading reservoirs, which can be functionalized with hyaluronic acid (HA) or other targeting agents to improve receptor-mediated uptake by cancer cells, as illustrated in Figure 5a [121,122,123].

Dendrimers, or dendritic macromolecules, have garnered significant interest owing to their highly branching, well-defined architectures that facilitate multivalent interactions with biological receptors. Their nanoscale, uniform characteristics and capacity for chemical functionalization render them optimal carriers for the targeted and accurate delivery of pharmaceuticals to cancer cells [124]. Moreover, liposomes and nanostructured lipid carriers (NLCs) offer complimentary approaches for the delivery of anticancer pharmaceuticals. Lipid-based systems, modified by polymers or modifying groups (e.g., HA targeting CD44), have good biocompatibility and can synergistically deliver multiple medications (e.g., baicalein and adriamycin for breast cancer treatment) [125,126].

In anticancer applications, these colloidal delivery systems depend on the precise adjustment of physicochemical parameters, including particle size, surface charge, and hydrophobicity. Colloidal systems possess stimulus-responsive components that react to particular environmental variables, like acidic pH levels in tumors and elevated enzyme concentrations. Surface modification techniques facilitate the attachment of certain ligands (e.g., folic acid, aptamers) to improve cellular absorption and minimize off-target effects [100,102,125]. The integration of these design principles has propelled the advancement of multifunctional targeted delivery systems anticipated to transform cancer therapy by improving efficacy, minimizing adverse effects, and promoting customized medicine.

Technologies for tumor-targeted delivery have emerged as a prominent research focus at the convergence of biomedical and food sciences. Food colloids, due to their distinctive physicochemical characteristics and biocompatibility, exhibit significant potential in the development of precise delivery methods. Polysaccharide-based (e.g., chitosan, pectin) and protein-based (e.g., whey proteins, zeinolysin) colloidal carriers can undergo surface modification to facilitate ligand-receptor-mediated target recognition. Alginate microcapsules exhibiting pH-responsiveness can facilitate over 90% regulated drug release in acidic circumstances that replicate the tumor microenvironment, offering a novel approach to mitigate the toxic side effects of chemotherapeutic agents on healthy tissues.

### 3.2. Brain-Targeted Delivery Systems

In recent years, food colloids have emerged as attractive carriers for brain-targeted delivery, owing to their biocompatibility, bioavailability, and intrinsic capacity to regulate drug release. Colloidal systems can be engineered to improve blood–brain barrier (BBB) permeability and facilitate targeted medication delivery to central nervous system (CNS) tissues by utilizing proteins like lactoferrin and casein, as well as food-derived macromolecules such as polysaccharides and lipids, including chitosan [3]. Food-grade materials provide inherent safety and regulatory compliance, exhibiting less immunogenicity and systemic toxicity relative to solely synthetic alternatives.

A crucial method in brain-targeted delivery systems aims to employ receptor-mediated transport across the blood–brain barrier (BBB). Lactoferrin, a naturally occurring protein in milk, can create biocoupled carriers with solid lipid nanoparticles (SLNs) for targeted brain administration by receptor-mediated endocytosis. Lactoferrin-conjugated solid lipid nanoparticles (SLNs) can enhance the targeting and prolonged release of anticancer agents, demonstrating increased cytotoxicity against brain cancers [105]. The creation of chitosan nanoparticles facilitates the direct trans-BBB transport of peptides and small bioactive molecules. Chitosan nanoparticles infused with therapeutic peptides can be preferentially transported to cerebral tissues with minimal hepatic and splenic absorption, underscoring their promise for non-invasive brain targeting [106].

The intranasal method of drug delivery has emerged as a compelling alternative for brain-targeted administration, leveraging the direct anatomical connection between the nasal cavity and the brain to circumvent the blood–brain barrier for precise drug delivery. Research indicates that colloidal carriers, including liposomes, nanoemulsions, and polymeric nanoparticles derived from food-grade substances, can be delivered intranasally to facilitate quick and effective drug delivery to deep brain regions while reducing systemic exposure [107,108]. This approach is especially advantageous in addressing neurodegenerative and acute central nervous system illnesses, where swift pharmacological intervention is necessary.

Moreover, new developments in multifunctional nanoparticle design emphasize surface changes and intelligent response architectures to accommodate the brain microenvironment. Multifunctional nanoparticles that integrate prolonged circulation characteristics with targeting moieties (such as peptides, aptamers, and carbohydrate ligands) have been engineered to surmount the blood–brain barrier. Food colloids can be similarly transformed into heterogeneous systems that encapsulate lipophilic anticancer or neurotherapeutic agents and respond to stimuli (e.g., pH, enzymes) within the brain parenchyma [109]. These alterations enhance drug release accuracy, diminish off-target effects, and enable in vivo imaging and monitoring, resulting in more tailored therapy strategies.

In summary, food colloids offer a flexible and promising foundation for brain-targeted delivery systems. They are constructed from natural food-grade components that provide intrinsic biocompatibility and safety, while advanced design techniques such as lactoferrin coupling, chitosan nanoparticle production, and intranasal administration improve blood–brain barrier penetration and facilitate targeted delivery to central nervous system tissues. The integration of food colloid science with nanotechnology is creating novel therapeutic opportunities for addressing brain disorders, including neurodegenerative diseases and brain tumors, with the potential for enhanced efficacy and less systemic toxicity.

### 3.3. Intestinal Targeted Delivery Systems

The gut serves as a primary location for nutrition absorption, and food colloids present advantageous platforms for targeted intestinal delivery by encapsulating bioactive substances, safeguarding them from the hostile gastric environment, and improving penetration across the intestinal mucus barrier. Food colloid delivery systems composed of food-grade proteins, polysaccharides, and lipids comply with regulatory safety criteria (GRAS status) and enhance drug breakdown and absorption in the small intestine.

A significant barrier in intestinal delivery is the passage of colloidal carriers through the mucus layer, whose permeability is influenced by mucus structure, age variations, and extracellular content. Subsequent research by Macierzanka et al. [127] and their team [128] demonstrated that the architecture of tiny intestinal mucus substantially influences the diffusion and transit of submicron particles. These studies indicate that the formulation of food colloids must consider the viscoelastic and structural properties of intestinal mucus to facilitate effective transit and intimate interaction with the epithelial surface.

The creation of food colloidal materials utilizing pH-responsive and enzyme-resistant substances might improve their capacity to safeguard and selectively release bioactives in intestinal environments. Sodium caseinate-stabilized zeinolysin colloidal particles have demonstrated efficacy as transporters of bioactive chemicals, functioning as a natural component that can form stable aggregates in aqueous conditions [76]. Moreover, pH-responsive systems can be engineered for the gastric environment (e.g., systems utilizing wormwood), which can facilitate regulated release by maintaining material integrity under acidic circumstances and disintegration under near-neutral conditions in the small intestine [110]. Wang et al. [3] established that these design principles can preserve the integrity and bioactivity of encapsulated components throughout gastrointestinal transit.

Alongside structural design tactics, programmable co-assembly methods have been employed to improve intestinal targeting. Figure 5b illustrates a study by Yang et al. [111] that revealed food-derived peptide co-assembly can improve targeted therapy for colitis by reducing degradation and retention issues associated with the intestinal barrier. This method proposes that the creation of carriers for natural biomolecules possessing self-assembling characteristics can enhance oral bioavailability and targeted medicinal efficacy.

The alteration of lipid-based colloidal carriers during intestinal digestion significantly influences medication solubilization and transport. Yao et al. [129] indicated that manufactured lipid nanoparticles can be dismantled and reassembled into mixed micelles within the gastrointestinal tract, hence enhancing the trans-intestinal epithelial transport of lipophilic bioactive substances. The self-assembly mechanism, facilitated by bile salts and phospholipids, can be utilized to enhance medication administration inside the dynamic intestinal milieu.

In conclusion, the investigation of food colloids in gut-targeted delivery systems necessitates the customization of the physicochemical characteristics of the carriers to address the specific constraints posed by the intestinal microenvironment. By targeting mucus permeability, pH responsiveness, and the dynamic rearrangement of lipid nanoparticles, these systems provide potential for improved protection, controlled release, and bioavailability of bioactive substances. These tactics underscore the potential of food colloids as multifunctional carriers for nutraceutical and pharmaceutical applications, thereby enhancing the efficacy of oral administration therapies.

### 3.4. Lung-Targeted Delivery Systems

Food colloids have arisen as prospective biocompatible carriers for lung-targeted delivery systems owing to their intrinsic safety, modifiable physicochemical features, and compatibility with inhalation delivery methods. In pulmonary medication administration, colloidal carriers can be designed as dry powders, aerosols, or solutions to improve deposition in the alveolar region of the lungs while addressing issues such as particle aggregation and fast clearance by alveolar macrophages [112].

The spray-drying process is a prevalent preparation method that transforms food-derived colloidal suspensions into nanostructured powders appropriate for dry powder inhalers (DPIs). Spray-dried nanoparticles can enhance aerodynamic qualities and ensure effective lung deposition, thereby enabling targeted administration of therapeutic medicines for respiratory conditions such as asthma and chronic obstructive pulmonary disease (COPD) [112]. These techniques can leverage the biosafety of food-grade components (e.g., proteins, lipids, polysaccharides), preserve colloidal stability, and enhance lung deposition efficiency.

A further interesting approach involves the production of nanoliposome suspensions. Liposomes originating from consumable lipids can be fabricated at nanoscale dimensions and generated as fine aerosols using nebulization. Research has highlighted that the physicochemical characteristics of liposome suspensions, such as particle size distribution and surface charge, are essential for aerosol stability and deposition patterns in the lungs [113]. Moreover, these nanosystems can encapsulate both hydrophilic and lipophilic pharmaceuticals, so offering dual functionality for localized targeting in the lungs and systemic administration via the alveolar epithelium.

Computational studies have clarified the interaction process between food colloidal nanoparticles and lung surfactant monolayers. Figure 5c illustrates the modeling of lipid-encapsulated gold nanoparticles to evaluate their transport kinetics via lung surfactant, yielding insights into colloidal stability and release dynamics at the alveolar interface [130]. Such investigations are crucial for optimizing the surface chemistry and dimensions of the carriers to avert aggregation with lung surfactant while facilitating effective medication release following deposition.

Moreover, specific chemical changes associated with L-cysteine or other targeting moieties have demonstrated enhanced pulmonary distribution. Research on L-cysteine-coupled poly-L-lactic acid nanoparticles incorporating 5-fluorouracil has demonstrated a marked enhancement in lung deposition, validating the function of L-cysteine in the pulmonary transport mechanism and indicating its prospective advantages in conditions like non-small cell lung cancer [114]. This indicates that integrating food-derived polymers with coupling techniques may efficiently leverage the inherent benefits of colloidal assemblies for lung-targeting applications.

Colloidal technologies, including solid lipid nanoparticles, polymer nanoparticles, and liposomes, are adaptable for lung-targeting applications [131]. The regulated dimensions, surface alterations, and responsive release mechanisms of these systems facilitate efficient deposition in the deep lung, enhance therapeutic efficacy, and diminish systemic adverse effects. Progress in aerosol engineering and particle size analysis can optimize these colloidal carriers to satisfy essential criteria for lung deposition while enhancing treatment efficacy [132].

In conclusion, the application of food colloids in lung-targeted delivery systems has been enhanced by a multidimensional strategy. Methods like spray drying and aerosol production have been refined to yield stable inhalable particles [112]. Computational and in vivo investigations have elucidated surface interactions with pulmonary surfactants and their subsequent dispersion within the pulmonary milieu [114,130]. As these systems advance, bespoke designs employing food-grade chemicals are expected to facilitate tailored therapy for respiratory illnesses, leveraging both topical and systemic treatment modalities while enhancing safety [131,132].

### 3.5. Skeletal Muscle-Targeted Delivery Systems

Food colloids present considerable potential as delivery methods targeting skeletal muscle, owing to their intrinsic biocompatibility, food-grade safety, and capacity for surface functionalization. These carriers, originating from natural macromolecules like proteins, polysaccharides, and lipids, can be engineered into nanoparticles, emulsions, or gels that encapsulate various therapeutic agents, including plasmid DNA and small-molecule drugs, enhancing biodistribution and enabling controlled release. Numerous studies indicate that surface modification using muscle-specific peptides markedly improves cellular uptake and transfection efficacy. Jativa et al. [133] demonstrated that a fifth-generation polyamidoamine dendrimer, modified with a skeletal muscle-targeting peptide (ASSLNIA), enhanced the delivery of plasmid DNA to mouse skeletal muscle cells, resulting in a three-fold increase in transfection efficiency compared to non-targeting vectors (illustrated in Figure 5d). Hersh et al. [134] created a multifunctional dendritic macromolecule nanocarrier adorned with a skeletal muscle targeting peptide (SMTP) and peptides that facilitate intracellular transport and nuclear localization, effectively tackling the obstacles of cellular uptake, endosomal escape, and nuclear entry in gene delivery. This research emphasizes dendrimer platforms, offering insights into design tactics for food colloids that could attain analogous muscle-specific targeting effects utilizing comparable food-grade ingredients, such as casein- or plant protein-based nanoparticles, alongside surface modification techniques.

Besides gene delivery, food-derived functional substances have been recognized as modulators of skeletal muscle. Chikazawa and Sato [115] illustrated that several naturally occurring dietary components function as β_2_-adrenergic receptor agonists, which are crucial for muscle protein synthesis and repair. The encapsulation of bioactive dietary components into colloidal carriers stabilizes them against gastrointestinal degradation and increases their effects on skeletal muscle by improving bioavailability. Moreover, Tacchi et al. [116] underscore the significance of bioengineered materials in muscle regeneration using scaffold biomaterials and nanotherapeutic methods, noting that the principles of material design and functionalization are parallel to those of food colloidal delivery systems.

Recent research by Hicks et al. [135] indicates that nanoparticles can be preferentially dispersed to regenerating skeletal muscle areas, including those affected by Duchenne muscular dystrophy (DMD). The biodistribution experiments underscore the promise of targeted delivery methods that integrate the superior biocompatibility and adaptability of food colloids with skeletal muscle targeting techniques (e.g., peptide coupling) to facilitate localized therapy while minimizing systemic side effects.

In conclusion, food colloids employed as carriers for targeted distribution to skeletal muscle signify a pioneering research domain that amalgamates the benefits of natural food-derived substances with sophisticated nanotechnology approaches. Utilizing surface modification techniques, such as muscle-targeting peptide coupling demonstrated in dendrimer systems and encapsulating biologically active food compounds with established anabolic functions, these carriers can proficiently surmount biological barriers and deliver therapeutic payloads to skeletal muscle tissue. Moreover, findings from scaffold-based muscle regeneration research [116] and biodistribution assessments of affected muscle [135] endorse the implementation of these design concepts in food colloid-based strategies for precision-targeted therapeutics for skeletal muscle disorders.

### 3.6. Heart-Targeted Delivery Systems

Food colloids are garnering heightened interest as efficient vehicles for cardiac-targeted delivery systems, particularly in the management of myocardial ischemia and infarction. Owing to their biocompatibility, inherent safety, and capacity for engineering from natural or food-grade lipids and biopolymers, such colloids can be customized to improve drug solubility, safeguard unstable bioactives, and facilitate preferential enrichment of ischemic myocardium via passive and active targeting mechanisms [117].

An exemplary case is polyethylene glycolated lipid nanoparticles encapsulating baicalein, a flavonoid possessing cardioprotective properties. Zhang et al. [117] demonstrated that these nanoparticles, characterized by their advantageous low nanoscale size, enhance pharmacokinetic profiles and promote transport to injured cardiac tissue. The efficacy was partially ascribed to the increased permeation retention (EPR) effect resulting from arterial rupture during acute myocardial ischemia, enabling nanoscale carriers to preferentially accumulate in the impacted area [117]. These studies demonstrate that food colloidal carriers derived from biocompatible lipid matrices can diminish systemic toxicity while enabling regulated, delayed medication release profiles aimed at cardiac treatments.

An alternative method involves active targeting through peptide-conjugated polymer nanoparticles. Sun et al. [118] documented the effective administration of miR-133 utilizing PEG-PLA nanoparticles decorated with RGD peptides. The RGD moiety is recognized for its binding affinity to integrin receptors that are overexpressed in cardiac tissues after infarct injury. This alteration substantially augments nanoparticle uptake into infarcted lesions, leading to a localized increase in therapeutic molecules and enhanced cardioprotection. The integration of polyethylene glycolization, a method to extend circulation duration, with receptor-mediated targeting demonstrates that food colloidal carriers can be engineered for accurate cardiac-targeted delivery via a dual mechanism.

Recently, vegetable oil-derived Pickering nanoemulsions have been examined as cytoplasmic drug delivery vehicles [119]. Their main objective has been to investigate non-endosomal drug transport; nevertheless, the durability of nanoscale size and stable food-grade particles offers a pathway for creating carriers that can circumvent endosomal breakdown and be tailored for cardiac targeting. Optimizing particle size, surface charge, and stability may enable Pickering nanoemulsions to promote direct cytoplasmic release of cardioprotective drugs in the heart, thereby diminishing clearance by immune cells and enhancing therapeutic payload [119].

In summary, our investigations indicate that food colloidal systems, such as polyethylene glycolized lipid nanoparticles, RGD-functionalized polymeric nanocarriers, and food-grade Pickering nanoemulsions, provide viable platforms for the targeted delivery of cardioprotective pharmaceuticals. By employing passive targeting through EPR effects and active targeting via receptor-mediated uptake, medication accumulation in the ischemic heart can be enhanced, pharmacokinetic stability can be augmented, and systemic side effects are anticipated to be reduced. Future research may concentrate on enhancing surface modification, particle stability, and release kinetics of these nanocarriers to attain improved precision in cardiac-targeted therapy.

### 3.7. Food Colloids in Application of Food Colloids in Oral Delivery Systems

Food colloids are pivotal in improving oral delivery by serving as biocompatible food-grade carriers that safeguard, transport, and enhance the absorption of various bioactive substances. These systems are generally composed of naturally occurring macromolecules such as proteins, polysaccharides, and lipids, and can be manipulated into emulsions, microemulsions, liposomes, and emulsion gels with regulated release characteristics. Benefits encompass increased solubility and stability of hydrophobic pharmaceuticals, gastrointestinal targeted protection, and augmented contact with intestinal epithelial cells [3,120].

Li et al. [120] revealed that a dietary polysaccharide-modified fish oil-based double emulsion formulation markedly enhanced peptide absorption by Caco-2 cells. The emulsion enhanced the oral absorption of peptides and indicated that such formulations may function as immunomodulators by promoting dendritic cell maturation and cytokine production. Wen et al. [136] indicated that water-in-oil microemulsions enhanced the preservation of L-glutathione against enzymatic degradation in the gastrointestinal tract, hence increasing its permeability across the intestinal epithelium. These investigations indicate that colloidal formulations can withstand the adverse conditions of the upper gastrointestinal tract and enhance effective medication delivery.

Wang et al. [3] offered significant insights into the physicochemical characteristics of food colloids and their dynamic release behavior, highlighting the critical role of carrier design in enhancing the nutritional value and organoleptic properties of functional foods while preserving the functional integrity of encapsulated bioactives. Zhang and McClements [137] highlighted that emulsion-based delivery systems can be customized to regulate the composition of gastrointestinal contents, thus influencing bioaccessibility and promoting improved absorption of hydrophobic nutraceuticals. This control is crucial for enhancing the oral bioavailability of isolated bioactives, rendering colloidal systems significant for both innovative medication formulations and improved whole food matrices.

Plant-derived colloidal delivery technologies enhance the versatility of these compositions. Tan and McClements [24] detailed the formulation of nanoemulsions, nanoliposomes, and microgels utilizing plant-derived components for the encapsulation and controlled release of oil-soluble vitamins, polyphenols, and carotenoids. The capacity to customize particle dimensions, internal architecture, and surface chemistry enables these systems to efficiently encapsulate and safeguard bioactive chemicals while reacting to gastrointestinal stimuli for precise release. Furthermore, Abdullah et al. [1] highlighted that emulsion gels, functioning as colloidal agents, can encapsulate bioactive compounds and affect digestion and absorption by altering texture and microstructure, hence offering novel methods for regulating nutrient release and uptake.

Perry and McClements [2] conducted a study on the oral administration of proteins and bioactive peptides, outlining current advancements in the encapsulating and safeguarding of bioactive proteins and peptides through food-grade colloidal systems. The discourse highlights that incorporating natural polymers into colloidal carriers can address issues of molecular instability and limited bioavailability, while facilitating controlled release in accordance with digestion kinetics.

In summary, the application of food colloids in oral delivery systems incorporates many design strategies to tackle significant problems associated with the gastrointestinal tract, such as enzymatic degradation, fluctuating pH levels, and the low solubility of numerous bioactives. These systems enhance the stability, absorption, and bioactivity of medicines and nutraceuticals by employing natural food-grade components and sophisticated preparation processes such as double emulsions, microemulsions, emulsion gels, and plant-based nano emulsions. This comprehensive strategy enhances the oral bioavailability of pharmaceuticals and facilitates the integration of immunomodulation and nutritional enhancement into functional food development.

The targeted distribution of food colloids to specific locations, including cancers, the brain, and the intestines, constitutes a key area of investigation in food nutrition and food materials science. This method seeks to improve the bioavailability of active constituents such as pharmaceuticals and minerals, facilitating tailored release. Food colloidal distribution encounters multiple hurdles, such as navigating significant pH gradient fluctuations, penetrating the intestinal mucosal barrier, and accommodating individual differences in gut flora. These concerns and challenges necessitate ongoing, comprehensive investigation in the future.

## 4. Research Progress on Food-Based Colloidal Systems for Nutrient Delivery

Recent years have shown substantial advancements in the application of food colloids for the exact targeted delivery of pharmaceuticals and bioactive chemicals, attributable to the designed adaptability of colloidal systems. Food colloids, including emulsion gels, protein nanoparticles, and colloids, provide various preparation techniques that can be tailored for regulated degradation, sensory experience, and precise delivery of food additives and therapies [1,2]. In food systems, the application of these colloidal carriers can enhance the solubility, stability, and intestinal absorption of encapsulated bioactives, while also improving the nutritional and functional attributes of the final product [3,4].

### 4.1. Food Colloids in Delivery of Vitamins and Minerals

Food-based colloidal systems have emerged as effective carriers for the tailored administration of vitamins and minerals, mitigating challenges such as inadequate solubility, low stability, and diminished bioavailability. Colloidal systems are generally constructed using food-grade natural macromolecules such as proteins, polysaccharides, and lipids as primary ingredients. They are engineered to encapsulate, safeguard, and regulate the release of water-soluble and fat-soluble vitamins and minerals, thereby augmenting their nutritional effectiveness in functional foods and beverages [3,38].

An essential technique entails employing plant-derived colloidal delivery systems. Tan and McClements [24] examined techniques for the fabrication of nanoemulsions, nanoliposomes, nanoparticles, and microgels exclusively from plant-based materials. Their research demonstrates that enhancing the composition, dimensions, surface chemistry, and internal architecture of these colloids can augment the solubility and chemical stability of oil-soluble vitamins, including vitamin D and precursors of vitamin A, as well as other lipophilic nutritional supplements. These technologies safeguard sensitive vitamins from oxidation and degradation during processing and storage, while offering regulated release patterns in the gastrointestinal tract. Maurya et al. [138] offered a unique design strategy for vitamin D, which has limited water solubility and poor chemical stability, by encapsulating it within nanoscale or micron-scale colloidal carriers to develop delivery systems appropriate for food and beverages. These delivery techniques address poor solubility challenges and improve bioavailability, preserving vitamin integrity until reaching absorption sites.

Correspondingly, microencapsulation techniques have been devised for the concurrent administration of vitamins and minerals. Gu et al. [139] documented egg white protein microgels infused with lipids, employing an injection gelation technique to encapsulate lipid droplets within a cross-linked protein matrix. These microgels not only safeguard the encapsulated lipid phase from degradation under gastrointestinal circumstances but also enhance the dissolution and absorption of fat-soluble vitamins and other micronutrients, hence increasing the functional value of the final product.

Moreover, investigations into colloidal delivery techniques respond to contemporary market requirements. Martins et al. [140] indicated that 27.1% of packaged foods for children in Brazil are enriched with essential vitamins and minerals such as vitamins A, B complex, C, D, calcium, iron, and zinc, underscoring the commercial significance of micronutrient fortification. Simultaneously, Shatnyuk et al. [141] established that innovative formulations—such as vitamin-mineral premix-enhanced bouillon cubes—substantially increase the vitamin concentration in reconstituted liquid diets. These market developments strongly encourage the development of colloidal carriers that safeguard, transport, and regulate micronutrient release in meals while preserving or improving sensory attributes. Strategies for fortifying staple foods such as bread may be enhanced by the use of micronutrient-enriched colloids [142]. This integration improves the nutritional profile of the final product and provides potential for customized physical and sensory attributes through the meticulous construction of colloidal structures.

In summary, advancements in food colloids for the delivery of vitamins and minerals encompass the creation of plant-based nanoemulsions and microgels for encapsulating lipophilic vitamins, as well as novel fortification techniques in processed foods. Employing natural materials can protect delicate micronutrients from deterioration, control their release, and eventually improve bioavailability. The integration of modern food colloid design and targeted fortification procedures in these delivery systems has considerable potential to enhance food nutritional quality and mitigate worldwide micronutrient shortages. Current challenges focus on clarifying the molecular interaction mechanisms between colloidal carriers and nutrients, improving product quality stability during large-scale production, and developing multi-nutrient synergistic delivery systems. Innovative Pickering emulsion colloidal systems and 3D printing techniques present new opportunities for the concurrent delivery of nutritional combinations, including B vitamins, vitamin E, and selenium. Future research will concentrate on creating intelligent colloids that can modulate gut microbiota to facilitate the dual integration of nutrition delivery and metabolic regulation.

### 4.2. Food Colloids in Encapsulation and Delivery of Probiotics and Bioactive Peptides

Food colloids have emerged as adaptable vehicles for the encapsulated delivery of probiotics in functional foods. The distribution of probiotics faces obstacles including hostile gastrointestinal environments, processing pressures, and storage conditions. Encapsulation with food-grade biopolymers, including proteins, polysaccharides, and lipids, creates a protective colloidal matrix that safeguards probiotics from acids, bile salts, and oxygen, while facilitating regulated release in the targeted intestinal area [143,144].

**Figure 6:**
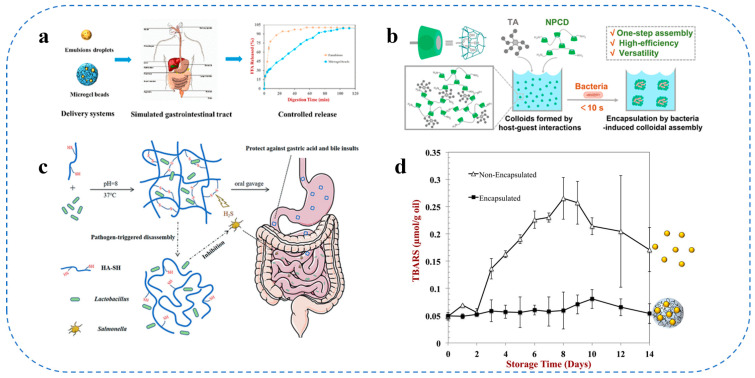
Food Colloids in Targeted Delivery of Nutrients (**a**) Protein microgels regulate lipid digestion [139]; (**b**) Bacteria-induced colloidal encapsulation technology for oral probiotics [145]; (**c**) Encapsulation of Lactobacillus rhamnosus in hydrogels for targeted pathogen delivery [147]; (**d**) Addition of caseinates as antioxidants, preparation of nano-emulsions and filled microgels for encapsulation and protection of omega-3 fatty acids [153].

A method involves bacterial-induced colloidal encapsulation, as illustrated in the research by Zhang et al. [145], wherein the viability of probiotics in simulated gastric fluid (pH 2.0 with pepsin) was markedly enhanced through the creation of a novel protective shell around the probiotic cells via NTc coating (depicted in Figure 6b). The technology employs the self-assembling characteristics of particular biopolymers to create a durable barrier akin to commercial enteric coatings, guaranteeing that an adequate quantity of viable cells reaches the gut. Complementary microencapsulation methods utilizing polymer matrices, such as polyvinylpyrrolidone-based polymer complexes, might facilitate the production of small particles to guarantee uniform probiotic dispersion while reducing effects on food texture [146,147]. These systems are explicitly engineered to sustain elevated cell counts (generally exceeding the 10^6^–10^7^ colony forming units (cfu)/g mandated by FAO/WHO criteria) and safeguard probiotic cells throughout processing and storage [148].

Another new strategy is the use of natural hydrogels for encapsulation. For instance, as seen in Figure 6c, Xiao et al. [147] encapsulated Lactobacillus rhamnosus within a hyaluronic acid-based hydrogel, which offered a barrier against water stress during cryogenic storage and improved intestinal-targeted delivery by facilitating pathogen-specific release. Hydrogel matrices, owing to their biocompatibility and modifiable cross-linking density, can preserve cell viability over prolonged storage and facilitate fast release under gastrointestinal circumstances. Li et al. [144] highlighted that microencapsulation techniques, such as ionic gelation or fluid-assisted procedures utilizing alginate [149], facilitate the maintenance of probiotic activity and enable meticulous regulation of capsule size and morphology. This control is crucial for ensuring the probiotic’s optimal activity and for reducing undesired organoleptic characteristics in the fortified food.

Advanced preparation techniques, such as microfluidic devices, enhance the encapsulation process by enabling fine control of particle size, shape, and encapsulation efficiency without language [150]. The delicate approach of microfluidics circumvents elevated temperatures and the production of substances that could compromise probiotic efficacy. Moreover, co-encapsulation technique, which involves the concurrent encapsulation of probiotics and prebiotics within dual-nucleated microcapsules, fosters synergistic effects and improves cellular stability and intestinal colonization efficacy. This dual-nucleus approach establishes distinct compartments for the probiotic and prebiotic substrates, safeguarding the probiotic cells during their transit through the stomach and enhancing development and bioactivity upon release.

Milk protein-based encapsulants, when utilized as a matrix, deliver nutrients while offering protection, making them suitable for integration into dairy products without modifying their intrinsic flavor or texture [151].

In summary, advancements in food colloids for probiotic encapsulation have produced several solutions that integrate natural polymer encapsulating technologies, sophisticated production techniques, and targeted release systems to address difficulties related to microbial viability. These colloidal systems augment the functional efficacy of probiotics in food applications, maintain elevated cellular activity from processing to ingestion, and play a crucial role in the advancement of the next generation of functional foods and nutraceuticals. Nevertheless, ongoing research continues to encounter numerous challenges, including the assessment of survival rates and stability of probiotics in the presence of gastric acid and digestive enzymes; the intestinal mucus layer and epithelial cell barrier obstructing the absorption of probiotics and bioactive peptides, consequently impacting delivery efficiency and targeting; and the elevated costs and intricate processes associated with large-scale production.

### 4.3. Food Colloids in Targeted Release of Functional Lipids

Food colloids have arisen as a viable approach for the precise delivery of functional lipids. Due to the frequent limitations of functional lipids, such as inadequate water solubility, vulnerability to oxidation, and low bioavailability, food colloid delivery systems have been created to encapsulate and safeguard functional lipids while also facilitating controlled and site-specific release profiles within the gastrointestinal (GI) tract. Nanolaminate coatings, solid lipid nanoparticles (SLNs), and biopolymer microgels have emerged as pivotal platforms for the targeted release of bioactive lipids, including ω-3 polyunsaturated fatty acids, carotenoids, and phytosterols [139,152]. The structure and function of bioactive substances are shown in Table 2.

A significant technique involves employing biopolymers to create nanolaminate coverings on lipid droplets. McClements delineates the architecture of these coatings, integrating several layers of biopolymers encasing droplets infused with lipophilic bioactives. Nanolaminate systems improve the stability of encapsulated lipids by resisting oxidative degradation and adjusting interfacial characteristics that regulate subsequent release. The multilayer construction facilitates a controlled release mechanism by allowing progressive diffusion of the lipid once the colloid arrives at the target region.

Moreover, solid lipid nanoparticles (SLNs) offer a flexible method for the delivery of bioactive lipids. Solid lipid nanoparticles (SLNs) are engineered to encapsulate lipophilic bioactives within a solid lipid matrix at room temperature [152]. The intrinsic stability of SLNs safeguards functional lipids against chemical degradation and enables careful regulation of their release kinetics throughout digestion. The regulated release is crucial for the efficient solubilization and absorption of functional lipids in the small intestine, thus enhancing their bioavailability. Another approach for encapsulating lipid droplets is the filled egg white protein microgels reported by Gu et al. [139]. In these systems, lipid droplets are dispersed within a network of crosslinked proteins to form a microgel that protects the lipids as they pass through the stomach. Upon arrival in the small intestine, pH changes and enzymatic digestion cause the microgel network to disintegrate, enabling controlled release of functional lipids. This method utilizes the natural gelation properties of egg white proteins to achieve targeted release while maintaining the organoleptic properties of the final food product.

The suppression of lipid oxidation is a crucial element influencing the release and bioactivity of functional lipids. Figure 6d illustrates that Chen et al. [153] established that the co-doping of casein with lipid droplets in nanoemulsions markedly diminished oxidative degradation. Casein functions as a natural antioxidant by stabilizing the emulsion structure during gastrointestinal transit, preserving the integrity of ω-3 fatty acids and facilitating their targeted release.

Furthermore, encapsulating techniques tailored for ω-3-rich fish oils have been investigated to address their instability and diminished bioavailability. Venugopalan et al. [154] examined various food-grade delivery strategies, including nanoemulsions and solid lipid nanoparticles (SLNs), that have effectively safeguarded ω-3 polyunsaturated fatty acids. These technologies augment cellular absorption and facilitate their integration into functional foods without detracting from the nutritional or sensory attributes of the product. Overall, these studies highlight the potential of food colloids in the targeted release of functional lipids. Controlled release is achieved by tailoring the structure and stability of nanolaminate coatings, SLNs and biopolymer microgels to maximize the bioavailability of lipophilic bioactive. The integration of such advanced colloidal delivery systems addresses the inherent limitations of functional lipids and paves the way for the next generation of functional foods and nutraceuticals capable of delivering targeted health benefits.

## 5. Applications of Nanoliposomes in Delivery of Bioactive Substances

Nanoliposomes, serving as multifunctional nanocarriers, have garnered significant interest for the transport of bioactive compounds in the pharmaceutical, nutraceutical, and food industries. Their distinctive architecture (bilayer lipid vesicles typically under 200 nm in diameter) can encapsulate both hydrophilic and lipophilic substances, offering benefits of biocompatibility, degradability, and possibility for controlled release. Moreover, the alteration of their physicochemical properties via surface modification, polymer coupling, or biopolymer coating can enhance their utility in targeted drug administration and precision nutritional formulations [155,156].

Nanoliposomes are extensively utilized for medication and gene delivery. Mozafari [155] conducted an extensive study of nanoliposome preparation and analytical methodologies, highlighting the significance of techniques like microfluidics in the creation of uniform nanoliposomes. These approaches not only attain excellent encapsulation efficiency for various bioactive chemicals, including anticancer agents and genetic material, but also provide intelligent stimulus-responsive release. Elkhoury et al. [156] observed that nanoliposomes may encapsulate various bioactive compounds and offer a regulated release profile, while their rapid clearance from systemic circulation can be mitigated through surface changes. Aguilar-Pérez et al. [157] reviewed the role of nanoliposomes as smart nanocarriers in biomedicine, emphasizing their safety and rapid therapeutic potential. The review showed that nanoliposomes hold great promise for encapsulating conventional drugs and delivering bioactive substances such as antioxidants and anti-inflammatory compounds. In the field of food science, Adeel et al. [158] demonstrated that nanoliposomes can encapsulate probiotics: encapsulation of Lactobacillus acidophilus with a specific polyelectrolyte enhances its activity under simulated gastrointestinal conditions, making it suitable for gut-healthy functional foods.

Targeted delivery is a significant application domain of nanoliposomes. Paolino et al. [159,160] indicated that conjugating thyroid stimulating hormone (TSH) to nanoliposomes led to a 3.5-fold enhancement in accumulation inside thyroid tissues relative to non-targeted systems. This receptor-mediated uptake approach illustrates how surface engineering might improve the tissue specificity of encapsulated pharmaceuticals. Zhang et al. [160] employed nanoliposomes to transport egg white peptide-calcium complexes, wherein hydrogen-bonding interactions facilitated the creation of amorphous dispersions of the active compounds, leading to a regulated release of calcium during digestion.

Nanoliposomes not only target specific organs but also enhance the bioactivity of polyphenolic substances effectively. Sun et al. [161] engineered hyaluronic acid-encapsulated nanoliposomes for the encapsulation of fexofenadine, a flavonoid characterized by low water solubility and poor stability. HA encapsulation has been demonstrated to preserve the spherical form of nanoliposomes while enhancing membrane fluidity, hence boosting cellular absorption and circulation duration. Rodriguez et al. [162] revealed that nanoliposomes formulated with rice bran phospholipids could effectively encapsulate quercetin (exceeding 84% efficiency) while preserving its chemical stability and biological activity over prolonged storage. A review by Rohilla and Dureja [163] summarized the application of nanoliposome formulations for the delivery of sensitive bioactive in cosmetics and food technology. For example, Aniya et al. [164] found that modification of nanoliposomes with inulin and pea proteins significantly improved thermal stability and controlled anthocyanin release during simulated digestion, thereby enhancing their sensory and nutritional properties in beverages such as hot cocoa.

Collectively, these advancements indicate that nanoliposomes serve as a dependable medium for the encapsulation and targeted distribution of various bioactive compounds. Nanoliposomes provide numerous benefits, including high encapsulation efficiency, controlled release kinetics, and enhanced bioavailability, whether employed in targeted drug or gene therapy in clinical environments, stabilization and release of sensitive nutrients in food systems, or improved skin delivery in cosmetic formulations. Future research is anticipated to concentrate on the issues associated with large-scale production and the further optimization of surface modification approaches for accurate targeting and extended in vivo circulation durations.

### 5.1. Preparation Methods and Advantages of Nanoliposomes

The formulation of nanoliposomes has evolved into a multifaceted study domain, with several preparation techniques providing benefits regarding particle size regulation, encapsulation efficacy, and product functionality. Traditional methods, including microfluidization, extrusion, and sonication, have been extensively employed to produce nanoscale liposome vesicles (typically under 200 nm in diameter) capable of encapsulating both hydrophilic and lipophilic substances.

Microfluidization employs high-pressure homogenization to amalgamate lipid and aqueous phases, resulting in uniformly sized liposomes suitable for large-scale production. Extrusion facilitates meticulous regulation of particle size distribution by directing a lipid slurry through a polycarbonate membrane with a predetermined pore size. Large-scale production can be burdensome [165]. Ultrasonication, particularly probe or bath sonication, is preferred for its operational simplicity and capacity to swiftly diminish vesicle size; yet it may be constrained by batch-to-batch variability and the risk of thermal degradation of sensitive bioactives. In his study, Mozafari [155] noted that nanoliposomes, as submicron-sized bilayer lipid vesicles, enable controlled release and cell-specific targeting by improving the water solubility and (in vitro and in vivo) stability of encapsulated bioactive. The high surface area of these nanoscale vesicles increases the interaction with biofilms, which in turn improves bioavailability and therapeutic efficacy. In addition, surface modification (e.g., polymer or ligand coupling) after formation optimizes targeted delivery and extends circulation time. This dynamic modification capability is one of the most significant advantages of nanoliposomes, driving their use in drug and gene delivery as well as nutraceutical and cosmetic applications.

Additional benefits of nanoliposomes encompass biocompatibility and degradability, acknowledged as safe, derived from natural phospholipids. Their capacity to encapsulate both hydrophilic and lipophilic molecules broadens the spectrum of deliverable active ingredients while safeguarding them against environmental influences such as oxidation, pH variations, and enzymatic degradation. Nanoliposomes possess controlled release characteristics and the capacity for targeted distribution via ligand coupling, enabling them to attain therapeutic concentrations at designated regions of action while reducing systemic side effects.

In conclusion, conventional preparation techniques including microfluidization, extrusion, and sonication provide distinct benefits for customizing nanoliposomes. Microfluidization and extrusion are essential for attaining uniform particle size and excellent encapsulation efficiency, whereas sonication offers a rapid and efficient method for particle size reduction. These methods, coupled with post-formulation modifications, facilitate the batch production of highly biocompatible nanoliposomes that improve solubility, stability, and permit controlled and targeted delivery of bioactive compounds, thereby advancing the pharmaceutical, nutraceutical, and cosmetic sectors.

### 5.2. Protective Role of Nanoliposomes for Unstable Bioactive Substances

Food colloids frequently safeguard unstable active ingredients by one or more of the following mechanisms: (1) Fully encapsulating unstable active compounds within carriers (e.g., liposomes, nanoemulsions, solid lipid nanoparticles) to establish a physical barrier that entirely isolates them from the external environment; (2) Leveraging confinement effects to disperse and immobilize active compounds within protein/polysaccharide gels, thereby restricting their molecular mobility and diffusion rates to decelerate degradation kinetics; (3) Utilizing controlled environments, such as pH or moisture levels, to protect substances sensitive to pH variations or susceptible to hydrolysis. Nanoliposomes, as biocompatible self-assembled bilayer vesicles, effectively safeguard unstable bioactives by separating sensitive chemicals from detrimental environmental influences, including oxidation, light, enzyme degradation, and heat instability. Encapsulating bioactive compounds within lipid bilayers enhances chemical stability and preserves bioactivity, hence prolonging shelf-life and enhancing the bioavailability of several nutraceuticals and pharmaceuticals [166,167].

A primary benefit of nanoliposome encapsulation is its capacity to safeguard extremely unstable chemicals, including those prone to oxidation and destruction. For instance, β-carotene, which is susceptible to light and oxidation, was encapsulated in nanoliposomes using high-speed homogenization, a technique that markedly diminished its breakdown rate while improving cellular absorption [166]. Volatile and degradable antimicrobial essential oils derived from oregano have been effectively encapsulated in small single-compartment nanoliposomes, enhancing the physical stability of the volatile compounds and regulating release profiles while preserving biological efficacy [167]. A separate study demonstrated that oligomeric proanthocyanidin molecules encapsulated in nanoliposomes exhibited a significant inhibitory effect on melanogenesis and safeguarded skin epithelial cells from UV-induced oxidative damage, indicating that lipid bilayers may function as a protective barrier against external stressors [168].

Subsequent research developments have concentrated on the surface modification of nanoliposomes to improve protection. Nanoliposomes containing fexofenadine encapsulated with hyaluronic acid (HA) impede the mobility of phospholipid head groups, thereby diminishing micropolarity and improving storage and thermal stability while maintaining the membrane fluidity of the hydrophobic core [161]. Chitosan-coated nanoliposomes were engineered to encapsulate walnut angiotensin-converting enzyme (ACE) inhibitory peptides, with the chitosan layer enhancing encapsulation efficiency and offering spatial stabilization to safeguard the bioactive peptides during storage and gastrointestinal transit [169].

In addition, the protective effect of nanoliposomes has been enhanced in formulations designed to co-encapsulate synergistic bioactive compounds. For example, lecithin-based nanoliposomes co-encapsulating serpentinone and xanthohumol achieved high encapsulation efficiency and significant antioxidant activity, suggesting that such multicomponent nano-formulations can protect the active ingredient from degradation while achieving controlled release [170]. Nanoliposomes with associated systems (e.g., tocopherol liposomes) can be effective in protecting sensitive bioactive from undesirable interactions in complex food and biological matrices, thereby enhancing stability and bioavailability [82,171].

In conclusion, the protective effects of nanoliposomes on unstable bioactives are numerous. For instance, encapsulating chemicals like β-carotene, essential oils, proanthocyanidins, and bioactive peptides within stabilized lipid vesicles can avert premature degradation of these unstable actives and guarantee continuous release. Surface modification using biopolymers like hyaluronic acid and chitosan enhances the protective effect by enhancing physicochemical stability and customizing release kinetics. Nanoliposomes serve as a dependable medium for the administration of sensitive bioactives in food, nutraceutical, and pharmaceutical applications, thereby enhancing efficacy and prolonging shelf life.

### 5.3. Application of Nanoliposomes in Oral Delivery of Peptides, Proteins, Polysaccharides, and Other Substances

Nanoliposomes have garnered significant interest as oral delivery systems for delicate biomolecules, including peptides, proteins, and polysaccharides. Their bilayer architecture offers dual compartments for encapsulating hydrophilic substances within water-soluble cores and lipophilic compounds in lipid bilayers, safeguarding unstable substances from adverse conditions like acidic pH in the gastrointestinal (GI) tract and enzymatic degradation, while also enhancing solubility and bioavailability following oral administration [172].

The research conducted by Sarabandi and Jafari [172] demonstrated that nanoliposomal carriers may efficiently encapsulate bioactive peptides derived from flaxseed. The insertion of peptides modified the zeta potential of the liposomes, suggesting that the low molecular weight peptide components interacted efficiently with the phospholipid monolayer. The alterations in surface charge may influence peptide stability, mucosal adherence, and subsequent absorption in the gastrointestinal tract, thereby improving their oral bioavailability. This outcome underscores the necessity of customizing the surface characteristics of nanoliposomes to improve the transport of protein hydrolysates and peptide constituents.

Yokota et al. [173] delineated the application of lyophilized liposomes formulated with non-purified soy lecithin for the microencapsulation of casein hydrolysate. The bioactivity of the protein hydrolysate was retained during oral processing, enabling the final product to uphold satisfactory organoleptic characteristics. The lyophilization technique, along with adequate lipid selection, guaranteed that the nanoliposomes possessed excellent particle size and structural integrity for effective transit within the gastrointestinal system. The liposomes improve the stability and absorption of bigger bioactive proteins against degradation.

Auwal et al. [174] conducted a comparative analysis of the physicochemical stability and bioefficacy of Lipoid S75-based biopeptide nanoliposome complexes synthesized by standard procedures vs. those produced via direct heating. The findings indicated that the preparation process was essential for particle size distribution, polydispersity index, and encapsulation efficiency. While the nanoliposomes produced by both techniques demonstrated adequate stability under storage circumstances, the direct heating method exhibited a superior stability index under prolonged storage. The prolonged release of encapsulated biopeptides within these nanoliposomes enhances intestinal absorption, highlighting their effectiveness in oral administration applications.

This study illustrates the substantial benefits of nanoliposomes as an oral delivery mechanism for many bioactive substances. Their capacity to safeguard sensitive molecules by encapsulation, provide regulated release mechanisms, and improve mucosal adhesion through surface modification renders them especially appealing for the advancement of functional foods and nutraceuticals. The scalability of techniques like high-pressure extrusion and direct heating enhances their economic viability.

**Figure 7:**
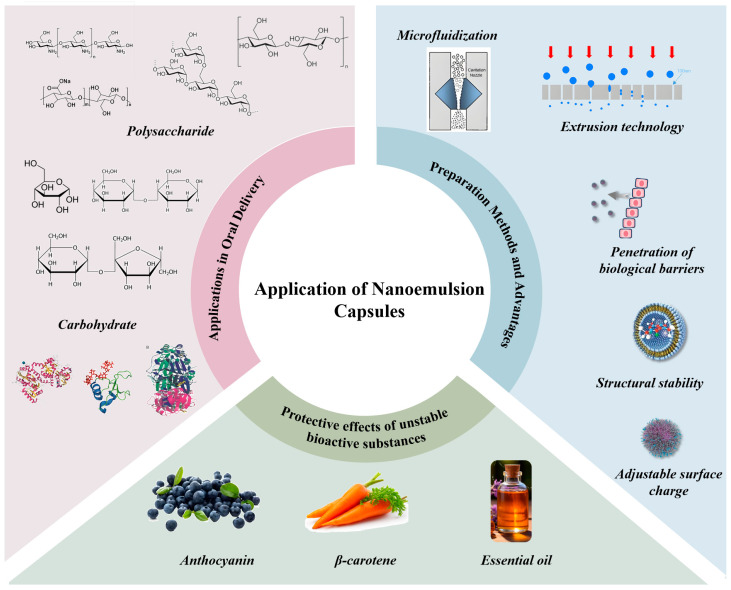
Application of nanoemulsion capsules.

In summary, the incorporation of nanoliposomes in oral delivery systems resulted in effective encapsulation and safeguarding of peptides, proteins, and polysaccharides, markedly enhancing their stability and bioavailability within the gastrointestinal milieu. The investigation of flaxseed-derived bioactive peptides, casein hydrolysates, and biopeptide complexes involved the modulation of parameters such as surface charge, particle size, and release kinetics, demonstrating the adaptability of nanoliposomes as vehicles for drug and nutrition delivery. Future research focused on optimizing production techniques and investigating surface functionalization strategies is anticipated to improve their applicability in nutritional supplementation and therapeutic interventions (Figure 7).

## 6. Patents and Commercially Available Products Based on Food Gels

### 6.1. Patents Based on Food-Derived Colloidal Carriers

Research patents on food-derived colloidal delivery systems predominantly employ components from food sources (such as proteins, polysaccharides, and lipids) to fabricate standard colloidal carriers (e.g., emulsions, liposomes, gel particles, and complexes). These provide the precise distribution of pharmaceuticals or bioactive agents to designated locations (e.g., stomach, small intestine, colon) through oral administration. This method improves the stability of pharmaceuticals or active compounds while enhancing the effectiveness of targeted distribution. Principal advantages encompass superior biocompatibility and environmental responsiveness (e.g., pH- or enzyme-mediated release). Table 3 encapsulates contemporary research patents pertaining to food-derived colloidal carriers.

### 6.2. Commercially Available Products Based on Food-Grade Colloids

Investigations into food-grade colloidal carriers have been commercialized as dietary supplements, functional foods, or medicinal excipients, having food-grade colloidal technology as their foundation. These products facilitate “targeted delivery” capabilities, specifically in colonic administration and improved bioavailability. Table 4 encapsulates the presently commercialized technology and products.

## 7. Conclusions

Colloidal targeted delivery systems based on food can selectively transport medications or nutrients to designated locations by altering the physicochemical characteristics of carriers and changing their surfaces with specific ligands, hence improving bioavailability and therapeutic effectiveness. This review encapsulates advancements in the application of food colloidal materials for the development of targeted delivery systems, emphasizing their roles in anticancer drug delivery, brain-targeted delivery, and intestinal-targeted delivery systems. Recent breakthroughs in the administration of bioactive components and the application of nanoliposomes for the oral transport of biomolecules are also emphasized. Food colloidal materials have superior biocompatibility and safety, rendering them optimal carriers for the development of tailored delivery systems.

Nonetheless, food colloidal-focused delivery methods continue to encounter the following limitations: (1) Restricted assortment of carrier materials, complicating the fulfillment of diverse drug or nutrient delivery requirements; (2) the stability and targeting efficacy of particular ligands for surface modification require enhancement; (3) intricate preparation procedures obstruct large-scale production; (4) inadequate in vivo metabolic assessments and safety evaluations. Future research should concentrate on creating innovative food colloidal materials that exhibit improved biocompatibility, degradability, drug-loading capacity, and modifiability. Integrating multi-modal targeting strategies—such as active versus passive targeting and physical versus chemical targeting—can enhance delivery efficiency. Before clinical implementation, food-based colloidal targeted delivery systems must undergo comprehensive preclinical investigation and clinical testing. Preclinical evaluation must include pharmacokinetic, toxicological, and pharmacodynamic investigations to determine the in vivo performance, safety, and effectiveness of the delivery system.

Despite various challenges, the advancement of innovative food-grade materials, refinement of composite targeting strategies, enhancements in manufacturing processes, and comprehensive clinical translation research will augment the efficacy of food colloid-based targeted delivery systems in disease treatment and nutritional intervention. These technologies provide an innovative approach for accurate medicine and nutrient administration, showcasing extensive application potential.

## Figures and Tables

**Table 1:** Application of food colloids in precision targeted delivery systems.

Target Position	Design Strategy	Advantages	Reference
Tumors	Aptamer-based systems	Designed to target the acidic tumor microenvironment or external stimuli (e.g., light, temperature) to promote drug penetration, prolong circulation time, and achieve selective accumulation through enhanced permeation retention (EPR) effects	[100,101,102]
	Dendritic macromolecular system	Precise delivery of drugs directly to cancer cells through multiple interactions with cellular receptors	[102]
	Other colloidal systems (e.g., functionalized multi-walled carbon nanotubes (f-MWNTs))	Introduction of folic acid or polyethylene glycol modification enhances solubility, biocompatibility and tumor targeting ability	[103,104]
Brain	Utilization of a receptor-mediated trans-BBB transport system	Improved targeting and sustained release of anticancer drugs showing enhanced cytotoxicity against brain tumors	[105,106]
	Intranasal drug delivery system	Colloidal carriers such as liposomes, nanoemulsions, and polymeric nanoparticles prepared from food-grade materials can be administered intranasally to enable rapid and efficient drug transport to deep brain structures while minimizing systemic exposure	[107,108]
	Surface Modification and Smart Response Architecture Design for Multifunctional Nanoparticle Systems	Combining long-cycling properties with targeting moieties (e.g., peptides, aptamers, carbohydrate ligands) not only encapsulates lipophilic anticancer or neurotherapeutic drugs, but also responds to stimuli (e.g., pH, enzymes) in the brain parenchyma. These modifications improve the precision of drug release, reduce off-target effects, and facilitate in vivo imaging and monitoring	[109]
Intestinal tract	Development of food colloidal systems using pH-responsive and enzyme-resistant materials	Enhanced ability to protect and strategically release bioactives under intestinal conditions.	[110]
	Programmable Co-Assembly Methods	Food-derived peptide co-assembly enhances targeted therapy for colitis by minimizing degradation and retention challenges posed by the intestinal barrier	[111]
Lungs	Powder systems designed as nanostructured	Enhanced deposition in the alveolar region of the lung while overcoming challenges of particle aggregation and rapid clearance by alveolar macrophages	[112]
	Generation of nanoliposome suspension liquid systems	Encapsulates both hydrophilic and lipophilic drugs, thus providing dual functionality for local targeting in the lungs and systemic delivery through the alveolar epithelium	[113]
	Targeted chemical modifications coupled to L-cysteine or other targeting groups	Improved lung distribution and significant increase in lung deposition	[114]
Striated muscle	Encapsulation from plasmid DNA to small molecule drugs using carriers derived from natural macromolecules such as proteins, polysaccharides and lipids	Addressing cellular uptake, endosomal escape, and nuclear entry challenges in gene delivery	[3]
	Embedding of bioactive substances of food origin	Stabilizes against gastrointestinal degradation and enhances its effects on skeletal muscle by improving bioavailability	[115,116]
Heart	Polyethylene glycolized lipid nanoparticles	Reduced systemic toxicity while facilitating a controlled delayed drug release profile for cardiac therapies	[117]
	RGD-functionalized polymer nanocarriers	Significantly enhanced uptake of nanoparticles into infarcted lesions, resulting in increased local concentrations of therapeutic molecules and improved cardioprotection.	[118]
	Vegetable oil-based Pickering nanoemulsion	Promoting more direct cytoplasmic release of cardioprotective agents in the myocardium has the potential to reduce immune cell clearance and increase therapeutic payloads	[119]
Oral	Emulsions, microemulsions, liposomes and emulsion gels	Enhanced solubility and stability of hydrophobic drugs, gastrointestinal (GI) targeted protection, and improved interaction with intestinal epithelial cells	[3,120]

**Table 2:** Structure and Function of Bioactive Substances.

Classification	Structure	Function	Representative Substance
Functional Lipids	Triglycerides	Functional energy storage, providing essential fatty acids, and promoting the absorption of fat-soluble vitamins.	EPA/DHA
Carotenoids	Isoprene derivatives	Antioxidant, immunomodulatory, photoprotective	β-carotene, lutein, lycopene
Phytosterols	Cyclopentane-fused fully hydrogenated phenanthrene (Steron)	Lower cholesterol	β-sitosterol, stigmasterol, campesterol

**Table 3:** Patents for Food Colloidal Carriers.

Carrier System	Materials	Characteristics	Representative Patents
Protein Colloid Systems	Whey protein, casein, soy protein, zein, pea protein	Utilizing the amphiphilic nature of proteins and their self-assembly properties under specific conditions (such as pH changes, heating, or enzymatic digestion) to form nanoparticles or micelles.	WO2021174007A1
Polysaccharide Colloid Systems	Chitosan, Alginate, Pectin, Starch, Cellulose	Utilizing sodium alginate to form an egg-carton-like gel structure through Ca^2+^ cross-linking, which remains stable in gastric acid and swells and dissolves at the neutral pH of the intestine to release the drug. Chitosan can open the tight junctions of the intestinal epithelium to promote absorption.	EP2890396B1
Liposome Colloid Systems	Solid lipid nanoparticles (SLNs), nanostructured lipid carriers (NLCs), liposomes.	Using emulsifiers (such as lecithin + Tween) and homogenization processes, hydrophobic substances are encapsulated within a lipid core to facilitate their lymphatic absorption in the small intestine.	US2018002859A1
Composite Colloid Systems		Constructing colloidal systems such as emulsion gels/high-core-phase emulsions, lipid-polymer hybrid nanoparticles, and layer-by-layer self-assembled microcapsules to enhance drug stability and achieve targeted controlled release.	US2019032147A1

**Table 4:** Commercially Available Food Gels.

Technology	Raw Materials	Targeted Mechanism	Product	Application
Microemulsion	Fats, phospholipids, polyglycerides	Lymphatic absorption targeting, significantly enhancing bioavailability	Accucor^®^ (Lonza, Basel, Switzerland), VESIsorb^®^ (VesiFact, Zug, Switzerland)	CoQ10, Omega-3, vitamins, CBD, and other hydrophobic supplements
Polysaccharide Gel System	Alginate, pectin, chitosan	pH-triggered or microbial enzyme-triggered	Various probiotic products, COLAL^®^ (IFT Pharma, Berwyn, PA, USA)	Probiotics (intestinal colonization), prebiotics, colon disease medications
Enteric-coated system	Alginate	pH-triggered, targeted intestinal release	Eudragit^®^ (Evonik, Essen, Germany), ACRYL-EZE^®^ (Colorcon, Harleysville, PA, USA)	Loading ibuprofen and other medications
Protein-based embedding system	Whey protein, Casein, Zein	Physical protection, enhancing intestinal absorption	Various premium functional foods and supplements	Encapsulate active substances such as vitamins, minerals, and polyphenols

## Data Availability

No new data were created in this study. Data sharing is not applicable to this article.
